# The conscious processing of emotion in depression disorder: a meta-analysis of neuroimaging studies

**DOI:** 10.3389/fpsyt.2023.1099426

**Published:** 2023-06-28

**Authors:** Xin-yun Gou, Yu-xi Li, Liu-xue Guo, Jing Zhao, Dong-ling Zhong, Xiao-bo Liu, Hai-sha Xia, Jin Fan, Yue Zhang, Shuang-chun Ai, Jia-xi Huang, Hong-ru Li, Juan Li, Rong-jiang Jin

**Affiliations:** ^1^School of Health Preservation and Rehabilitation, Chengdu University of Traditional Chinese Medicine, Chengdu, China; ^2^Affiliated Hospital of Chengdu University of Traditional Chinese Medicine, Chengdu, China; ^3^Department of Rehabilitation, Mianyang Hospital of Traditional Chinese Medicine, Mianyang, China; ^4^Mental Health Center, West China Hospital, West China School of Medicine, Sichuan University, Chengdu, China

**Keywords:** depression, emotion processing, conscious, functional magnetic resonance imaging, activation likelihood estimation

## Abstract

**Background:**

Depression is generally accompanied by a disturbed conscious processing of emotion, which manifests as a negative bias to facial/voice emotion information and a decreased accuracy in emotion recognition tasks. Several studies have proved that abnormal brain activation was responsible for the deficit function of conscious emotion recognition in depression. However, the altered brain activation related to the conscious processing of emotion in depression was incongruent among studies. Therefore, we conducted an activation likelihood estimation (ALE) analysis to better understand the underlying neurophysiological mechanism of conscious processing of emotion in depression.

**Method:**

Electronic databases were searched using the search terms “depression,” “emotion recognition,” and “neuroimaging” from inceptions to April 10th, 2023. We retrieved trials which explored the neuro-responses of depressive patients to explicit emotion recognition tasks. Two investigators independently performed literature selection, data extraction, and risk of bias assessment. The spatial consistency of brain activation in conscious facial expressions recognition was calculated using ALE. The robustness of the results was examined by Jackknife sensitivity analysis.

**Results:**

We retrieved 11,365 articles in total, 28 of which were included. In the overall analysis, we found increased activity in the middle temporal gyrus, superior temporal gyrus, parahippocampal gyrus, and cuneus, and decreased activity in the superior temporal gyrus, inferior parietal lobule, insula, and superior frontal gyrus. In response to positive stimuli, depressive patients showed hyperactivity in the medial frontal gyrus, middle temporal gyrus, and insula (uncorrected *p* < 0.001). When receiving negative stimuli, a higher activation was found in the precentral gyrus, middle frontal gyrus, precuneus, and superior temporal gyrus (uncorrected *p* < 0.001).

**Conclusion:**

Among depressive patients, a broad spectrum of brain areas was involved in a deficit of conscious emotion processing. The activation of brain regions was different in response to positive or negative stimuli. Due to potential clinical heterogeneity, the findings should be treated with caution.

**Systematic review registration:**

https://inplasy.com/inplasy-2022-11-0057/, identifier: 2022110057.

## 1. Introduction

Depression is a highly prevalent psychiatric disorder. The age-standardized prevalence for depression was 30,814 cases per 100,000 people in 2019, and it is the second leading cause of years lived with disability (1). The incidence of mental health problems increased during the COVID-19 pandemic, especially in the initial phase of the outbreak. Since the pandemic, depression is three times more prevalent than before (2, 3). Clinical and sub-clinical depression are often complicated with cognitive function impairment (4), poor quality of life (5), and significant suicidal ideation (6). Worldwide, there are 1,253 million disability-adjusted life-years caused by mental disorders, of which depression accounts for up to 37.3% (1). In the United states, the incremental economic burden of adults with depression has been estimated to be as much as $326.2 billion including direct costs, suicide-related costs, and work productivity losses (7).

Conradi et al. (4) found that cognitive impairment presented in 94% of patients during depressive episodes. Emotion recognition is an important manifestation of cognitive function; it is the ability to identify multiple emotions in verbal or facial expression, which ensures effective social communication. Emotion recognition tasks are divided into explicit tasks and implicit tasks, which are two links in emotion recognition. During implicit tasks, stimuli features are collected and subjects are required to classify non-emotional information (such as color and sex). However, the process of implicit emotion recognition is too quick to be aware of. In contrast to passive emotion perception (implicit tasks), explicit tasks are usually considered as active and conscious processing of emotion. Different from the implicit process, explicit tasks require subjects to classify the emotion based on the stimuli features and prior knowledge (8). Conscious emotion recognition needs more cognitive effort and time, and it is more likely to be damaged (9–12). Current studies showed that depressive patients achieved less accuracy in emotion recognition tasks when compared with the healthy subjects (13, 14). Impairment in conscious emotion recognition increases the difficulty in maintaining interpersonal interactions, which makes people feel more exclusive from society and exacerbates depressive symptoms, even increasing suicide risk (13, 15, 16).

The abnormal conscious recognition of emotion in depression is associated with abnormal neural activations. Functional magnetic resonance imaging (fMRI) is a powerful and widely used tool to explore neurophysiological activity. Several studies investigated the neuro-mechanism of depression during explicit tasks based on fMRI (17–19). Li et al. (17) found that the abnormal activated brain regions including the dorsolateral prefrontal cortex and temporal gyrus were responsible for the altered performance during the explicit emotion task. In another study (18), the brain abnormalities primarily focused on the cerebellum, precentral gyrus, amygdala, insula, and others. Briceño et al. (19) discovered a decreased activation on the precuneus in depressive patients during emotion-recognition tasks. The aberrant brain activation related with depression remains incongruent. To resolve the inconsistency among studies, several meta-analyses have been conducted to explore the brain activities of emotion processing in depression (20–27). In the previous meta-analyses, both explicit and implicit tasks were included and synthesized. Critchley et al. (28) concluded that explicit tasks activated the temporal lobe cortex, and implicit tasks activated the amygdala region. Marrazzo et al. (29) proved that prefrontal areas, inferior parietal lobule, and high-level visual cortex played key roles in discriminating implicit/explicit tasks. Pierce et al. (30) took cerebellum as a region of interest, and discovered that posterior cerebellar hemispheres activated in response to explicit tasks, and bilateral lobules VI/Crus I/II, right Crus II/lobule VIII, anterior lobule VI, and lobules I-IV/V activated during implicit tasks. Since explicit and implicit tasks may have different neural activities, the brain activation in response to implicit or explicit tasks should be synthesized separately. Different from implicit tasks, the process of explicit emotion recognition requires the recruitment of further cognitive resources, and it is more likely to be impaired. In addition, the impairment of explicit emotion recognition is associated with depressive symptoms. Elucidation of brain activation related with defective conscious emotion recognition in depression can provide potential targets for the treatment of depression. Activation likelihood estimation (ALE) is a quantitative synthesis method for neuroimaging studies. In contrast with effect size, ALE pays more attention to effect location, calculates the likelihood of activation of each voxel, tests the stabilization of the activation across studies, and gets spatial consistency between studies. In this study, we planned to conduct ALE analysis to investigate the neurophysiological mechanisms of conscious processing in patients with depression during explicit tasks based on fMRI.

## 2. Methods

The ALE analysis has been registered on the International Platform of Registered Systematic Review and Meta-analysis Protocols (INPLASY) (https://inplasy.com/inplasy-2022-11-0057/) (Registration No. 2022110057). We reported this ALE analysis according to the PRISMA guidelines (31).

### 2.1. Literature search

**Figure 1:**
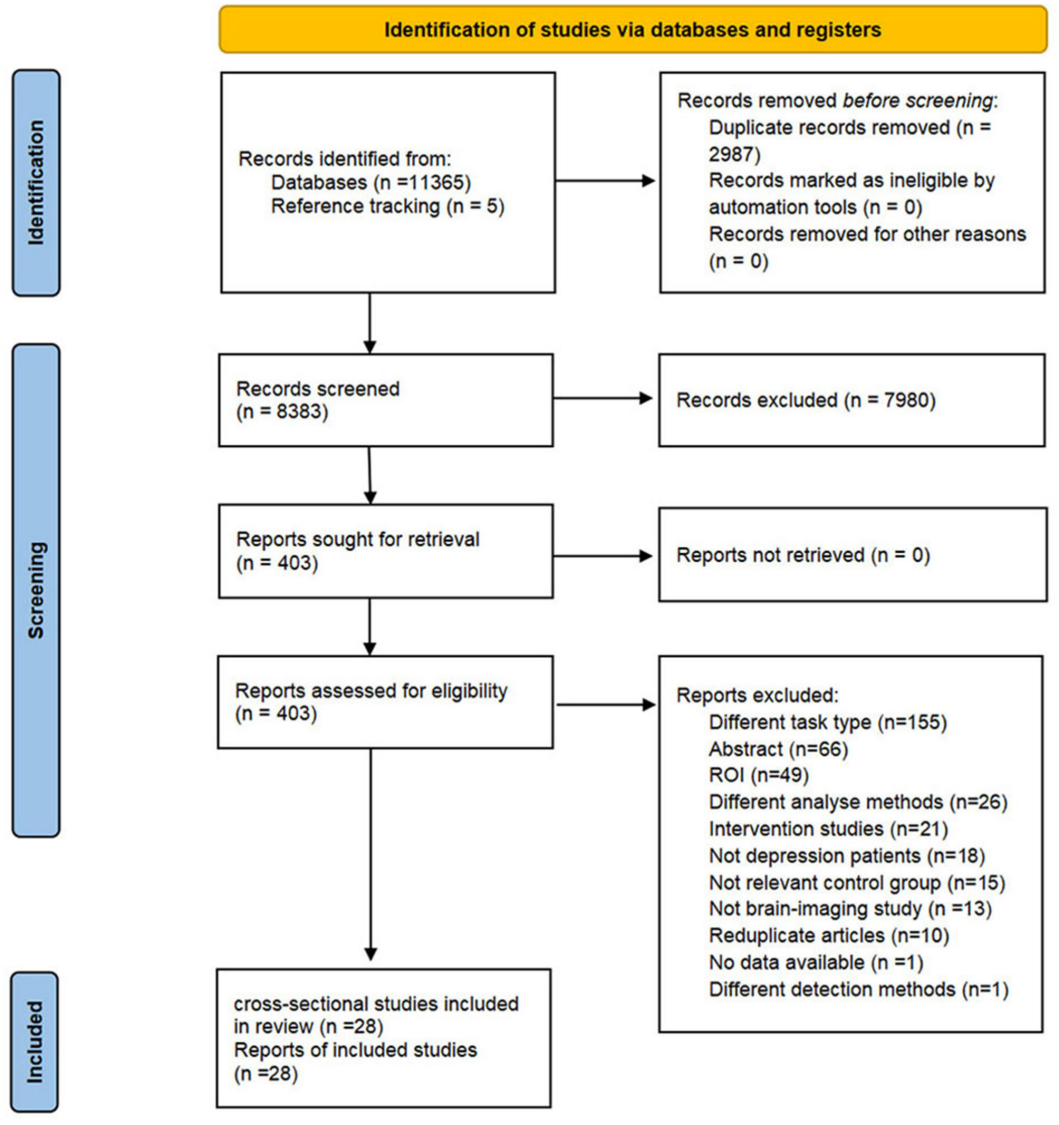
PRISMA flow chart.

The flow diagram of study selection is shown in Figure 1. Four English databases (Cochrane, Embase, Web of Science, and PubMed) and four Chinese databases [Chinese Biomedical Literature Database (CBM), China National Knowledge Infrastructure (CNKI), Wanfang databases, and Chinese Scientific Journal Database (VIP)] were systematically searched from inceptions to April 10th, 2023. We retrieved trials which explored the neuro-responses of depressive patients in response to explicit emotion recognition tasks. The search strategies were combined with Medical Subject Headings (MeSH) and free text words related to the following terms: depression, emotion recognition, and neuroimaging. We also reviewed the reference lists of relevant articles to identify additional literature. Detailed search strategies are provided in Supplementary Table 1.

### 2.2. Inclusion criteria

The pre-specified inclusion criteria were as follows:

A. Types of studies: cross-sectional studies published in Chinese or English which explored specific brain activity during emotion categorization, emotion perception, or emotion matching tasks in patients with depression.
B. Patients: patients with depressive episodes fulfilled the diagnostic criteria of the Diagnostic and Statistical Manual of Mental Disorders (DSM-IV/V) (32), International Classification of Diseases (ICD-10) (33), or Chinese Classification and Diagnostic Criteria of Mental Disorders (CCMD) (34).
C. Comparator: never depressed healthy participants matched to depressive patients.
D. Exposure: emotional faces/voices/rhythm were used as negative, positive, or positive/negative affective stimuli.
E. Outcome: the whole-brain coordinates were obtained by comparisons between depressive patients and never-depressive healthy participants; coordinate space was normalized to the Montreal Neurological Institute (MNI) or Talairach.

### 2.3. Exclusion criteria

Studies were excluded if they met any of the following criteria: (1) protocol, abstracts, review, or case report; (2) patients with bipolar I disorder, sub-threshold depression, postpartum depression or depression secondary to brain injury, Parkinson's Disease, Alzheimer's Disease, etc.; (3) no details of diagnostic criteria; (4) duplicated articles or overlapping subjects; (5) imaging was performed via structural imaging, magnetic resonance spectroscopy, or functional near-infrared spectroscopy; (6) resting-state, brain functional connectivity, or brain activation-functional correlations studies; (7) studies focused on regions of interest analysis; or (8) data could not be extracted.

### 2.4. Study selection

We imported retrieved records into Endnote software (X9), and duplicates were removed. Two reviewers (JZ and XYG) independently screened titles and abstracts to exclude irrelevant records. Then, the remaining records in the full text were scrutinized according to pre-specified eligible criteria. In case of overlapping samples between studies, we retained the study with a larger sample size. Any disagreement was resolved by team discussion until a consensus was reached.

### 2.5. Data extraction

Two independent reviewers (XBL and DLZ) extracted data with a pre-defined data extraction form. The extracted data included study characteristics (author, publication year, sites), participant information (sample size, sex ratio, age, diagnostic criteria, and subtypes and severity of depression), task performance (task paradigms and stimulus types), fMRI acquisition and analysis (fMRI acquisition, MRI processing, and software version) and results of imaging (spatial coordinates of vertices and three-dimensional coordinates system).

After extraction, two reviewers cross-checked data to ensure accuracy. Any discrepancy was arbitrated by the third reviewer (JL).

### 2.6. Risk of bias

Two reviewers (YXL and HSX) independently assessed the risk of bias of included studies using the modified version of the Newcastle–Ottawa scale (NOS) (35, 36) adapted to fMRI. The tool contains 11 methodological items which are categorized into 4 domains (selection, comparability, exposure, and statistical analysis). If the item was addressed properly, it was rated as “+”; “–” indicated that the condition was not met; and “?” meant that the information was not clear or missing. Referring to the study of Gentili et al. (36), the specific criteria is shown in Supplementary Table 2. A third author (RJJ) supervised the entire process to resolve discrepancies.

### 2.7. Statistical analysis

This ALE analysis was performed with Ginger-ALE 2.3.6 software (www.brainmap.org/ale). Talairach coordinates were converted into MNI coordinates using Ginger-ALE 2.3.6; all coordinates were presented in the MNI space. We used cluster-level family wise error (FWE) (cluster *p* = 0.05, 1,000 permutations, uncorrected *p* = 0.001) to explore the spatial consistency of overall increased activity and decreased activity, respectively. Additionally, we used the Ginger-ALE users' manual as the reference and conducted uncorrected *p*-value with a conservative threshold (uncorrected *p* < 0.001), and removed clusters under a user-chosen size by setting the Min. Volume as 250 mm^3^ to find additional overlap and decrease the possibility of false-negatives. MRIcron and a common anatomical template in MNI space (ch2bet.nii.gz) were used for visualizing the results. We conducted two overall analyses of emotion processing based on the brain activation direction across negative and positive stimuli.

### 2.8. Subgroup analysis

Subgroup analyses were conducted based on different stimulus types (negative and positive), age of the participant (age ≦ 18 and age > 18), and severity of depression.

### 2.9. Sensitivity analysis

A whole-brain voxel-based jackknife sensitivity analysis was performed to test the robustness of the results by removing one dataset each time.

### 2.10. Qualitative analysis

The significant differential clusters of explicit tasks between patients with depression and healthy participants were reported in all studies. Each abnormal brain region was extracted to calculate the number of replicates in the overall analysis, negative stimuli analysis, and positive stimuli analysis, separately.

## 3. Results

### 3.1. Screening results of studies

In total, 11,365 articles were retrieved from eight databases. We identified 8,383 records after removing duplicates, and reference tracking yielded five additional records. After screening the titles and abstracts, 403 articles were retained for full-text reading. As a result, we included 28 eligible cross-sectional studies. The detailed selection procedure is shown in Figure 1. The reasons for exclusion are listed in Supplementary Table 3.

### 3.2. The characteristics of included studies

The characteristics of included studies are shown in Table 1. Among 28 included studies, 20 studies (17–19, 40–47, 49, 50, 52, 53, 55, 57–59, 61) were published in English and 8 (37–39, 48, 51, 54, 56, 60) were in Chinese. A total of 656 patients with depression and 680 healthy subjects were involved. The average age of participants ranged from 15 to 66 years old, and only 2 studies (40, 55) focused on youths with depression. Depressive patients in 20 studies (17–19, 40–47, 49, 50, 52–55, 57, 59, 61) were diagnosed according to the DSM, 1 (37) in the light of CCMD, and 2 (51, 58) based on ICD; 3 studies applied DSM-IV and CCMD-3 (38, 39, 60); 1 used both DSM-IV and ICD-10 (56); 1 adopted CCMD-3 and ICD-10 (48). Hamilton Depression Rating Scale, Beck Depression Inventory, Montgomery–Asberg Depression Scale, Center for Epidemiologic Studies Depression Scale, and Children's Depression Rating Scale-Revised were adopted to screen depression. Tasks used in included studies were classified into the following three categories: emotion categorization (37, 46, 51, 52, 54, 59), emotion matching task (40–42, 44, 47, 57, 61), and emotion perception (17–19, 38, 39, 43, 45, 48–50, 53, 55, 56, 58, 60) (including valence assessment, emotion judgment, and elicited emotion rating). 6 studies (17–19, 40, 50, 55) performed tasks with alternation of positive stimuli and negative stimuli, the results were the reflection of overall task performance; 20 studies (37–39, 41–49, 51, 54, 56–61) analyzed negative stimuli; and 11 studies (37–39, 43, 46, 49, 52, 53, 56–58) focused on positive stimuli. As for imaging tools, 21 studies (17, 18, 40–43, 46, 48–61) acquired images on a 3T scanner, and 6 studies (37–39, 44, 45, 47) on a 1.5T scanner. 21 studies (17, 18, 37–39, 42–47, 49–54, 56, 58–60) used SPM software for pre-processing and analysis, three studies (40, 48, 55) used AFNI software, and 2 studies (41, 61) used FSL software. The details of fMRI acquisition and analysis are shown in Supplementary Table 4.

**Table 1:** The characteristics of included studies.

**References**	**Sample size**		**Age of depression (y, mean ±SD)**	**Depression severity (mean ±SD)**	**Diagnostic criteria**	**Task paradigm**	**Coordinate space**	**Depression subtype**	**Stimuli used in study**	**Activated brain areas**
	**Depression (F/M)**	**HC (F/M)**								
Li (37)	15 (9/6)	23 (13/10)	29.07 ± 11.3	HAMD: 24.53 ± 5.2	CCMD-3	Emotion categorization	Talairach	MDD	Negative/Positive	Positive: Middle frontal gyrus, medial frontal gyrus, middle temporal gyrus, angular gyrus, Lingual gyrus, Precuneus, inferior/Superior parietal lobule, cuneus, fusiform gyrus, thalamus, putamen, middle occipital gyrus, insula Negative: inferior frontal gyrus, inferior occipital gyrus, Middle frontal gyrus, insula, anterior cingulate cortex, parahippocampal gyrus, superior/middle/inferior temporal gyrus, precuneus, inferior/Superior parietal lobule, cerebellum, midbrain
Yao (38)	18 (18/0)	18 (18/0)	40.1 ± 11.5	HAMD: 50.1 ± 8.7	DSM-IV, CCMD-3	Emotion perception	Talairach	MDD	Negative/Positive	Positive: Middle frontal gyrus, Superior parietal lobule, superior temporal gyrus, postcentral gyrus Negative: Middle frontal gyrus, supramarginal gyrus, middle occipital gyrus, Precuneus
Cao et al. (39)	12 (0/12)	12 (0/12)	43 ± 11	HAMD: 46 ± 8	DSM-IV, CCMD-3	Emotion perception	Talairach	MDD	Negative/Positive	Positive: middle occipital gyrus, Middle/inferior frontal gyrus, precentral gyrus, inferior parietal lobule, postcentral gyrus, superior/middle temporal gyrus, declive Negative: Middle frontal gyrus, cingulate, fusiform gyrus, postcentral gyrus
Yang et al. (40)	12 (5/7)	12 (5/7)	15.9 ± 1.4	CDRS-R: 76 ± 9	DSM-IV	Emotion matching	Talairach	MDD	NA	Parahippocampal, anterior cingulate, cingulate gyrus, cuneus
Townsend et al. (41)	15 (9/6)	15 (9/6)	45.6 ± 11.2	HAMD: 20.1 ± 4.9	DSM-IV	Emotion matching/labeling	MNI	MDD	Angry or fearful	Insula, medial temporal gyrus, inferior temporal gyrus, occipital gyrus, lingual gyrus, hippocampal gyrus, putamen, cerebellum
Scheuerecker et al. (42)	13 (10/3)	15 (10/5)	37.9 ± 10.1	HAMD: 20.5 ± 4.7	DSM-IV	Emotion matching	MNI	MDD	Angry or fearful	Middle frontal gyrus, precentral gyrus, caudate nucleus, supplementary motor area, precuneus
Derntl et al. (43)	15 (9/6)	15 (9/6)	34.1 ± 11.95	HAMD: 19.9 ± 7.30	DSM-IV	Emotion perception	MNI	MDD	Happy/Angry	Positive: Calcarine, inferior occipital Gyrus, lingual Gyrus, Cerebellum, Amygdala Negative: Cerebellum, fusiform gyrus, inferior frontal gyrus, lingual gyrus superior occipital gyrus
van Wingen et al. (44)	18 (11/7)	48 (31/17)	33.3 ± 11.7	HAMD: 21.8 ± 4.2	DSM-IV	Emotion matching/labeling	MNI	MDD	Angry or fearful	Anterior cingulate cortex, inferior frontal gyrus, amygdala, intraparietal sulcus, inferior occipital gyrus, putamen and insula, middle occipital gyrus, precuneus
Ritchey et al. (45)	11 (8/3)	7 (5/2)	36.1 ± 10.1	BDI: 25.1 ± 8.8	DSM-IV	Emotion perception	MNI	MDD	Negative	Anterior temporal lobe, DLPFC, insula, dorsal ACC, VLPFC, superior frontal sulcus, medial frontal gyrus, precentral gyru, hippocampus, superior/inferior temporal gyrus
Schlund et al. (46)	10 (6/4)	10 (5/5)	46.7 ± 7.2	HAMD: 25.5 ± 6.2	DSM-IV	Emotion categorization	Talairach	MDD	Angry/Happy/Sad	Negative: Culmen, parahippocampus, insula, superior temporal, claustrum, inferior parietal, caudate, medial frontal, thalamus, middle frontal, anterior cingulate Positive: Amygdala, lingual gyrus, precuneus, posterior cingulate, middle temporal, thalamus, putamen, culmen, anterior cingulate, middle frontal, caudate, inferior frontal, parahippocampus, inferior parietal, cuneus
Zhong et al. (47)	27 (16/11)	25 (14/11)	20.37 ± 1.86	CES-D: 25.11 ± 5.42	DSM-IV	Emotion matching	MNI	MDD	Angry or fearful	Amygdala, inferior temporal gyrus, middle/Superior frontal gyrus, thalamus
Tu (48)	6 (5/1)	8 (5/3)	33 ± 6.62	HAMD: 26.17 ± 5.6	CCMD-3, ICD-10	Emotion perception	Talairach	MDD	Negative	Negative: Middle/superior frontal gyrus, Middle occipital gyrus, anterior cingulate cortex, middle/inferior temporal gyrus, fusiform gyrus, parahippocampal gyrus, Lingual gyrus, putamen
Li et al. (49)	42 (26/16)	35 (19/16)	32.4 ± 10.1	HAMD: 24.26 ± 6.47	DSM-IV	Emotion perception	MNI	MDD	Negative/Positive/Neutral	Negative: Superior/middle/inferior frontal gyrus, precentral gyrus, superior temporal gyrus, inferior parietal lobule, precuneus, supramarginal gyrus, anterior/posterior cingulate cortex, cerebellum, postcentral gyrus, middle/inferior occipital gyrus, cuneate lobe, lingual gyrus, fusiform gyrus Neutral: Medial/middle frontal gyrus, superior/medial temporal gyrus, precuneus, cerebellum, caudate nucleus, cingulate cortex, precentral gyrus, superior /middle/inferior occipital gyrus, lingual gyrus, fusiform gyrus, middle temporal gyrus
										Positive: Superior/medial frontal gyrus, hippocampal gyrus, precentral/postcentral gyrus, superior temporal gyrus, precuneus, middle occipital gyrus
Lisiecka et al. (50)	A: 30 (19/11) B: 20 (14/6)	A: 21 (11/10) B: 25 (13/12)	A: 40.7 ± 9.0 B: 45.7 ± 12.5	HAMD: A: 29.0 ± 6.5 B: 28.1 ± 6.6	DSM-IV	Emotion perception	MNI	MDD	NA	Precuneus/posterior cingulate cortex, rolandic operculum, insula, angular gyrus, supramarginal gyrus
Bian (51)	33	20	38.7 ± 9.1	HAMD: 45.3 ± 6.90	ICD-10	Emotion categorization	MNI	MDD	Negative	Superior temporal gyrus, Precuneus, Posterior Cingulate, cerebellum, angular gyrus
Briceño et al. (19)	A: 15 B: 12 C: 12 D: 14	A: 19 B: 13 C: 12 D: 13	A: 29.2 ± 7.8 B: 25.5 ± 3.5 C: 64.8 ± 6.3 D: 66.0 ± 9.6	HAMD: A: 17.4 ± 4.3 B: 14.8 ± 4.4 C: 16.3 ± 6.3 D: 14.6 ± 4.4	DSM-IV	Emotion perception	MNI	DD	NA	Precuneus
Skokauskas et al. (52)	A: 32 (25/7) B: 13 (6/7)	43 (23/20)	A: 42.27 ± 11 B: 38.61 ± 8	BDI: A: 32.16 ± 11.3 B: 38.38 ± 9.17	DSM-IV	Emotion categorization	MNI	MDD	Positive/ neutral	Positive: Angular, supramarginal, inferior orbital frontal, mid temporal, Neutral: Fusiformis
Murrough et al. (53)	18 (8/10)	20 (9/11)	38.1 ± 13.8	MADRS: 29.9 ± 6.8	DSM-IV	Emotion perception	MNI	MDD	Happy	Insula, caudate
Cai et al. (54)	18 (11/7)	21 (13/8)	28.3 ± 6.2	HAMD: 29.9 ± 6.9	DSM-IV	Emotion categorization	MNI	MDD	Negative/Neutral	Negative: Medial frontal Neutral: Medial/inferior frontal gyrus, anterior cingulate cortex
Ho et al. (55)	26 (19/7)	37 (23/14)	16.1 ± 0.3	BDI: 28.4 ± 2.0	DSM-IV	Emotion perception	MNI	MDD	NA	Medial prefrontal cortex, posterior cingulate cortex, parahippocampal cortex, amygdala, lentiform nucleus, anterior insula, inferior frontal gyrus, middle temporal gyrus, fusiform gyrus, lingual gyrus, middle occipital cortex
Xu (56)	A: 12 (7/5) B: 12 (5/7)	A: 12 (7/5) B: 12 (5/7)	A: 32.67 ± 6.3 B: 29.83 ± 6.97	HAMD: A: 20.58 ± 2.02 B: 23.67 ± 2.57	DSM-IV, ICD-10	Emotion perception	MNI	DD	Negative/Positive	Negative: fusiform gyrus, superior temporal gyrus, insula, Precuneus, middle/superior frontal gyrus, cuneus, thalamus, supramarginal gyrus, paracentral lobule, precentral gyrus Positive: superior/middle frontal gyrus, superior/middle temporal gyrus, Middle occipital gyrus, insula, superior parietal lobule, angular gyrus
Bürger et al. (57)	36 (22/14)	36 (19/17)	40.72 ± 11.58	HAMD: 23.61 ± 3.17	DSM-IV	Emotion matching	MNI	UD	Happy/angry/fearful	Positive: Putamen, caudate nucleus, pallidum, insula, hippocampus, thalamus, superior/middle temporal gyrus, amygdala, rolandic operculum, gyrus temporalis tranversi, postcentral gyrus, frontal inferior operculum, Superior/medial frontal gyrus, cingulate gyrus Negative: cingulate gyrus, medial/superior orbital frontal gyrus, Superior/middle frontal gyrus
Mel'nikov et al. (58)	21	21	NA	NA	ICD-10	Emotion perception	MNI	DD	Negative/Positive	Positive: Middle/upper frontal gyrus, precentral gyrus, lingual gyrus, fusiform gyrus Negative: Middle/upper frontal gyrus, frontal operculum, postcentral gyrus, precentral/postcentral gyrus, frontal operculum, supramarginal gyrus, middle/upper temporal gyrus, cerebellum
Groves et al. (59)	16 (10/6)	10 (4/6)	35.4 ± 12.7	MADRS: 22.1 ± 9.3	DSM-IV	Emotion categorization	MNI	MDD	neutral, posed sad and genuine sad	Negative: Medial orbital frontal, Putamen, caudate, medial frontal/orbitofrontal cortex, dorsolateral prefrontal cortex, anterior cingulate, posterior cingulate, superior parietal lobe, lingual gyrus, cuneus
Koch et al. (18)	30 (16/14)	30 (14/16)	29.9 ± 8.9	BDI: 29.5 ± 8.3	DSM-IV	Prosody emotion perception	MNI	MDD	NA	Superior/middle temporal gyrus, cerebellum, precentral gyrus, amygdala, inferior frontal gyrus/insula, postcentral gyrus
Song et al. (60)	14 (9/5)	14 (8/6)	20–60	HAMD: 17–24	DSM-IV, CCMD-3	Emotion perception	MNI	DD	Negative	Middle/inferior temporal gyrus, Middle frontal gyrus, inferior parietal lobule
Nagy et al. (61)	21 (14/7)	21 (14/7)	32.52 ± 9.55	BDI: 23.10 ± 5.66	DSM-5	Emotion matching	MNI	MDD	Negative	Accumbens, subcallosal cortex, anterior paracingulate gyrus, precentral/postcentral gyrus, inferior parietal lobule, precuneus, middle occipital gyrus, fusiform gyrus
Li et al. (17)	37 (25/12)	37 (19/18)	31.21 ± 12.17	HAMD:20.11 ± 6.895	DSM-IV	Voice emotion perception	MNI	MDD	NA	Ventromedial/dorsolateral prefrontal cortex, angular gyrus, middle occipital gyrus, inferior temporal gyrus

HC, healthy control; F, female; M, male; HAMD, Hamilton Depression Rating Scale; BDI, Beck Depression Inventory; MADRS, Montgomery–Asberg Depression Scale; CES-D, Center for Epidemiologic Studies Depression Scale; CDRS-R, Children's Depression Rating Scale-Revised; DSM-IV, Diagnostic and Statistical Manual of Mental Disorders; ICD-10, International Classification of Diseases; CCMD, Chinese Classification and Diagnostic Criteria of Mental Disorders; MNI, Montreal Neurology Institute; MDD, Major Depressive Disorder; UP, unipolar depression.

### 3.3. Risk of bias assessment

The detailed information of each included study is shown in Supplementary Table 5. The selection part contained two items for included patients and healthy controls respectively. In the domain of case definition, the diagnosis of all studies was based on standard criteria, which was considered as “+”. As for the representativeness of the cases, there were 3 studies (38, 51, 54) that included only women or men, which was judged as “–”; and 1 study (58) was rated as “?”, because it did not provide information on the sex of included subjects; a defined procedure for inclusion was reported in the remaining studies, which were appraised as “+”. In the selection of controls, 13 studies reported that the healthy controls were recruited from the same community of individuals with depression; while 6 studies (17, 43, 48, 50, 52, 53) got “–” because the sources of healthy subjects were different from depressive patients; 9 studies (37, 39, 42, 45–47, 51, 58, 60) did not state the origin of the healthy subjects, and were determined as “?”. With reference to definition of controls, psychiatric assessment of healthy subjects were not conducted in 2 studies (43, 53), were judged as “-”, and 7 (19, 38, 41, 42, 45, 47, 58) did not specify the screening tool for healthy subjects, thus were considered as “?”.

Regarding the comparability of studies, it contained two aspects: age/gender and other variables. Among 26 studies, the age and gender were matched between depression and healthy controls, which were regarded as “+”; 2 studies (46, 59) did not mention the resemblance of age or sex between the two groups, then were considered as “?”. As for the comparability of other variables, 6 studies (41, 45–47, 53, 59) were rated as “?” because they did not provide the comparability of other demographic variables, the remaining studies were all reported clearly, and were rated as “+”.

For the exposure, all the included studies reported that the same experimental procedure was performed in depressive patients and healthy subjects, thus were considered as “+”. Eleven studies reported the rate and causes of drop-out, and were rated as “+”; 15 trials (17, 18, 38–40, 43, 46, 48, 50, 52, 55, 56, 58, 60, 61) did not report the drop-out rate and relevant reasons, thus were evaluated as “-”; and 2 studies (51, 53) reported drop-out rate without explanation, then were rated as “?”. All the studies used behavioral tasks which could cause a significant difference between depressive patients and healthy controls, which were judged as “+”.

In the statistical analysis part, *p*-value > 0.001 was reported in 7 studies (40, 41, 44, 47, 51, 53, 54), which were regarded as “–”; 13 studies (17–19, 37, 43, 45, 50, 52, 55–57, 60, 61) without the description of uncorrected thresholds were considered as “?”; the remaining studies were classified as “+”. 10 (37–39, 42, 45–47, 49, 51, 59) out of 28 studies were rated as “–” due to a lack of false positive correction; 2 studies (56, 57) performed false positive correction without detailed description, and were appraised as “?”.

### 3.4. Meta-analytic of brain imaging

#### 3.4.1. Aggregated results

**Figure 2:**
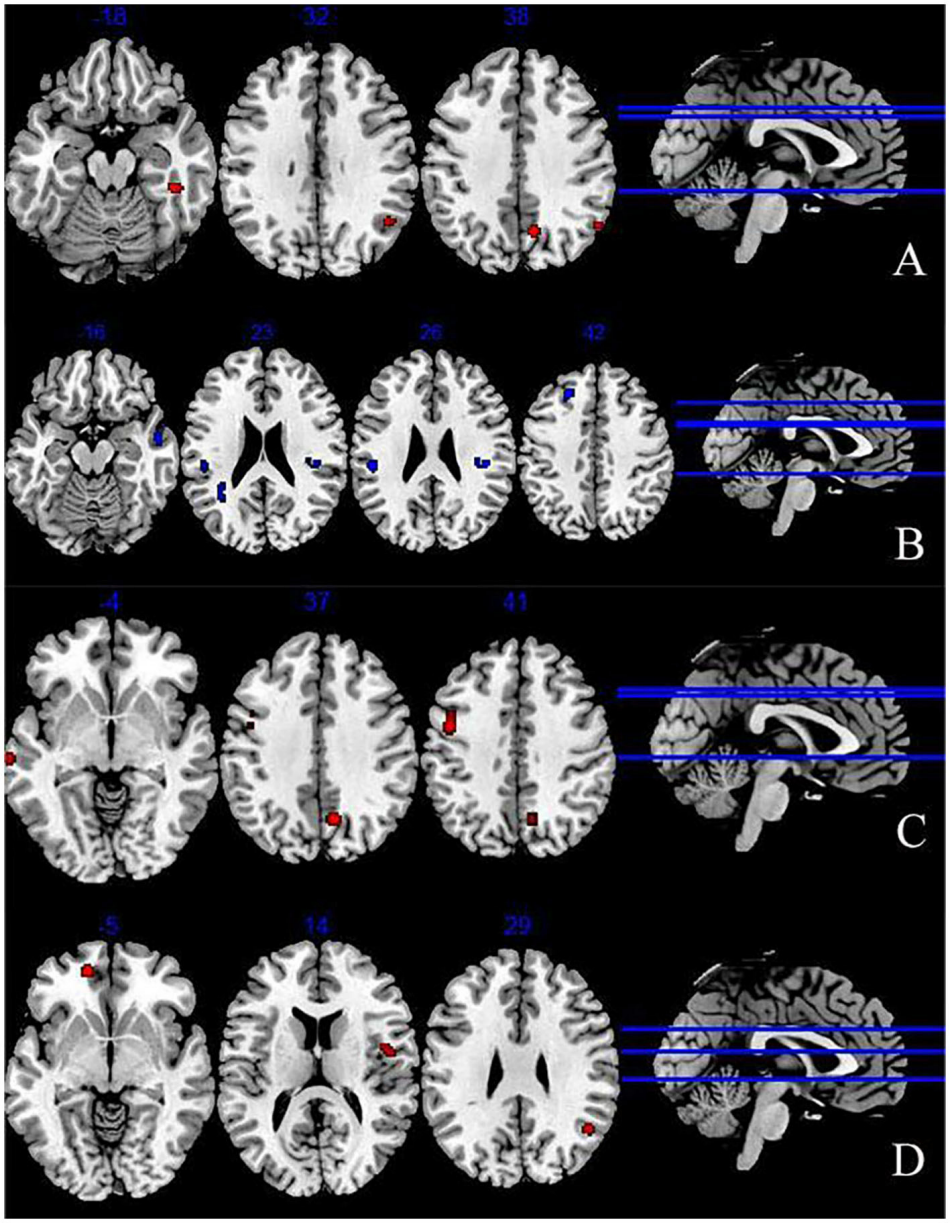
Abnormal brain regions during explicit emotion processing **(A)** increased brain regions during explicit emotion processing; **(B)** decreased brain regions during explicit emotion processing; **(C)** activated brain regions in response to negative stimuli; **(D)** activated brain regions in response to positive stimuli.

We extracted 193 increased foci from 24 studies (37–39, 42–49, 51, 52, 54, 56, 58–61) and 199 decreased foci from 24 studies (37–39, 41, 43, 44, 46–49, 52, 53, 56–58, 61). The overall analysis of explicit emotion recognition tasks showed an increased activity of the middle temporal gyrus (MTG) (BA 39), superior temporal gyrus (STG) (BA39), parahippocampal Gyrus (PHG) (BA36), and cuneus (BA7) in patients with depression (Figure 2A), and a decreased activity of STG (BA 22), inferior parietal lobule (IPL) (BA 40), insula (BA 13), and superior frontal gyrus (SFG) (BA 8) (Figure 2B). The results are shown in Table 2. However, no peak activation was detected when switching to the FWE-corrected level.

**Table 2:** Results of meta-analysis.

**Cluster number**	**Brain areas**	**R/L**	**MNI**			**Cluster volume (mm^3^)**	**ALE value (× 10^−2^)**	**Brain areas**	**BA**
			**x**	**y**	**z**				
**Main results**									
**Depression > healthy**									
1	Temporal lobe	R	48	−56	30	360	1.25	Middle temporal gyrus	39
2	Temporal lobe	R	54	−58	36		1.21	Superior temporal gyrus	39
3	Limbic lobe	R	46	−34	−16	336	1.40	Parahippocampal gyrus	36
4	Occipital lobe	R	10	−64	38	272	1.49	Cuneus	7
**Depression < healthy**									
1			−34	−46	22	352	1.30	No gray matter found	
	Temporal lobe	L	−34	−52	22		1.30	Superior temporal gyrus	22
2	Temporal lobe	R	54	−6	−14	312	1.34	Superior temporal gyrus	22
3	Parietal lobe	L	−48	−28	26	304	1.44	Inferior parietal lobule	40
4	Sub-lobar	R	44	−26	24	296	1.31	Insula	13
	Sub-lobar	R	36	−24	26		1.12	Insula	13
5	Frontal lobe	L	−20	32	42	256	1.30	Superior frontal gyrus	8
**Negative stimuli**									
**Depression > healthy**									
1	Frontal lobe	L	−44	−2	42	536	1.43	Precentral gyrus	6
	Frontal lobe	L	−44	6	40		0.98	Middle frontal gyrus	6
2	Parietal lobe	R	10	−62	38	384	1.49	Precuneus	7
3	Temporal lobe	L	−66	−22	−2	352	1.21	Superior temporal gyrus	21
**Positive stimuli**									
**Depression > healthy**									
1	Frontal lobe	L	−14	50	−4	376	1.29	Medial frontal gyrus	10
2	Temporal lobe	R	48	−56	28	336	1.11	Middle temporal gyrus	39
3	Sub-lobar	R	50	−6	12	264	1.02	Insula	13
	Sub-lobar	R	46	0	14		0.92	Insula	13
**Adults**									
**Depression > healthy**									
1	Occipital lobe	R	10	−64	38	304	1.49	Cuneus	7
**Depression < healthy**									
1			−34	−46	22	432	1.30	No gray matter found	
	Temporal lobe	L	−34	−52	22		1.30	Superior temporal gyrus	22
2	Sub-lobar	R	44	−26	24	424	1.31	Insula	13
	Sub-lobar	R	36	−24	26		1.12	Insula	13
3	Parietal lobe	L	−48	−28	26	368	1.44	Inferior parietal lobule	40
4	Frontal lobe	L	−20	32	42	352	1.30	Superior frontal gyrus	8
5	Parietal lobe		22	−64	46	264	1.17	Precuneus	7
	Parietal lobe	L	32	−66	48		1.11	Superior parietal lobule	7
**Adolescent**									
**Depression > healthy**									
1	Limbic lobe	L	−2	−51	15	384	0.83	Posterior cingulate	29
2	Anterior lobe	L	−4	−51	−2	352	0.86	Culmen	
3	Limbic lobe	R	18	34	10	288	0.68	Anterior cingulate	32
4	Limbic lobe	L	−24	−36	36	280	0.70	Cingulate gyrus	31
5	Sub-lobar	L	−18	18	20	272	0.69	Caudate	
6	Temporal lobe	L	−26	−46	8	256	0.67	Hippocampus	
**Depression < healthy**									
1	Sub-lobar	L	−33	21	0	288	0.83	Insula	13
2	Occipital lobe	L	−22	−88	−10	264	0.89	Fusiform gyrus	19
3	Occipital lobe	L	−42	−78	6	264	0.89	Middle occipital gyrus	19
4	Sub-lobar	R	27	−7	5	256	0.81	Lentiform nucleus	
**Severe depression**									
**Depression < healthy**									
1	Limbic lobe	R	28	−10	−20	320	0.77	Amygdala	
2	Frontal lobe	R	44	54	18	304	0.79	Superior frontal gyrus	10
3	Parietal lobe	R	42	−30	42	304	0.79	Inferior parietal lobule	40
4	Parietal lobe	R	20	−42	74	272	0.80	Postcentral gyrus	7

BA, Brodmann area; MNI, Montreal Neurological Institute.

#### 3.4.2. The ALE results of negative stimuli

The results of 100 activation foci from 15 experiments (37–39, 42, 44–49, 51, 54, 56, 60, 61) and 95 deactivation foci from 14 experiments (37, 39, 41, 43, 44, 46–49, 56–58, 61) demonstrated that patients with depression exhibited significant hyperactivity in the precentral gyrus (BA6), middle frontal gyrus (MFG) (BA6), precuneus (BA7), and STG (BA21). Results are presented in Figure 2C and Table 2. However, no significant result was found in the analysis of decreased activity in response to negative stimuli.

#### 3.4.3. The ALE results of positive stimuli

As shown in Figure 2D and Table 2, when depressive patients focused on positive stimuli, the synthesized results [67 activation foci of 10 experiments (37–39, 43, 46, 49, 52, 56, 58, 59) and 63 deactivation foci of 10 experiments (37, 38, 43, 46, 49, 52, 53, 56–58)] indicated that the activated clusters were centered at the medial frontal gyrus (BA10), MTG (BA39), and insula (BA13).

#### 3.4.4. The ALE results of subgroup based on age

We acquired 158 activation foci from 18 experiments (18, 38, 39, 42–46, 48, 49, 51, 52, 54, 56, 58–61) and 162 deactivation foci from 18 experiments (17, 19, 38, 39, 41, 43–46, 48–50, 52, 53, 56–58, 61) in depressive patients aged over 18. We found an increased activation in the cuneus (BA 7) and a decreased activation in STG (BA 22), insula (BA 13), IPL (BA 40), SFG (BA 8), precuneus (BA 7), and superior parietal lobule (BA 7).

In terms of depressive patients aged younger than 18, 7 activation foci and eight deactivation foci were found in 2 experiments (40, 55). The posterior cingulate (BA 29), culmen, anterior cingulate (BA 32), cingulate gyruse (BA 31), caudate, and hippocampus were activated, while insula (BA 13), fusiform gyrus (BA 19), middle occipital gyrus (BA 19), and lentiform nucleus were deactivated.

#### 3.4.5. The ALE results of subgroup based on severity of depression

Among 28 studies, 3 studies (38, 39, 51) analyzed the brain imaging of patients with severe depression, 5 studies (44, 45, 53, 54, 60) included patients with moderate to severe depression, and 20 studies recruited mild to severe depressive patients. We found 24 activation foci and 8 deactivation foci from the 3 studies. The brain activation of the amygdala, SFG (BA 10), IPL (BA 40), and postcentral gyrus (BA 7) reduced in patients with severe depression.

### 3.5. Sensitivity analysis

The jackknife sensitivity analysis results of the decreased activity revealed that the STG was replicable in all combinations; the inferior parietal lobule and superior frontal gyrus remained significant in 20 out of 22 combinations; insula sustained significance in 19 out of 22 combinations. The sensitivity analysis of increased activity showed MTG, STG, and PHG were significant in all but two combinations; the cuneus presented significance in all but three combinations. Details are shown in Supplementary Table 6.

### 3.6. Qualitative analysis results

**Figure 3:**
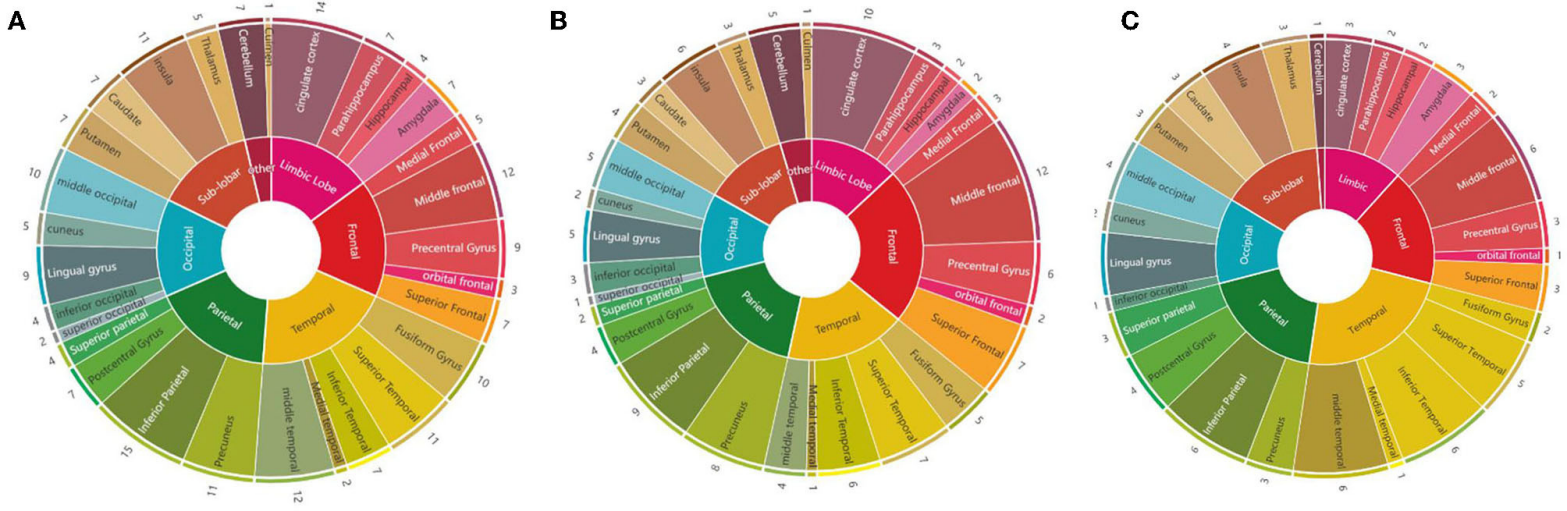
Summary diagrams of activated brain regions in the reviewed fMRI studies. **(A)** Mixed (negative, positive, and neutral) emotion recognition; **(B)** negative (sad, angry, and fearful) emotion recognition; **(C)** positive (happiness) emotion recognition.

We calculated the times that each brain region was mentioned in the 28 studies: the inferior parietal cortex was the most mentioned brain region (15 times), followed by the cingulate cortex (14 times), MFG (12 times), MTG (12 times), precuneus (11 times), insula (11 times), STG (11 times), middle occipital cortex (10 times), fusiform gyrus (10 times), and precentral gyrus (9 times). Considering the abnormal brain activation areas in response to negative stimuli, the top 10 most mentioned brain regions were MFG (12 times), cingulate cortex (10 times), IPL (9 times), precuneus (8 times), STG (7 times), superior frontal cortex (7 times), insula (6 times), inferior temporal cortex (6 times), precentral gyrus (6 times), and fusiform gyrus (6 times). In the positive stimuli, MFG (6 times), inferior temporal cortex (6 times), MTG (6 times), IPL (6 times), STG (5 times), insula (4 times), middle occipital cortex (4 times), precentral gyrus (4 times), cingulate cortex (3 times), and amygdala (3 times) were mentioned most. The results of each dataset are shown in Table 1 and presented in Figure 3.

## 4. Discussion

### 4.1. The activity changes of brain regions in response to explicit emotion processing in depression

Psychoradiology is an interdisciplinary subject of radiology and psychosis, which uses imaging techniques to detect the structural or functional activity of the brain in healthy populations or in people with psychiatric disorders (62–65). It breaks through the traditional diagnostic methods (scale assessment and subjective judgment of clinicians) and performs quantitative analysis to observe the subtle changes in the brain which is able to optimize the diagnosis, treatment, and monitoring of psychiatric disorders (62). In the present study, to investigate the brain activation changes in response to explicit emotion processing tasks, we pooled the results from 28 neuroimaging studies and the results indicated that people with depression had abnormal brain activities in contrast to healthy subjects during the explicit emotion recognition tasks. The results showed that MTG (BA39), STG (BA39), PHG (BA36), and cuneus (BA7) displayed a greater likelihood of hyperactivity, while the IPL (BA 40), bilateral STG (BA 22), insula (BA 13), and SFG (BA 8) exhibited hypoactivity.

We mapped these brain regions to functional networks according to the Stanford Atlas of Functional ROI. As for activated

brain regions, default mode network [DMN, including PHG, STG (BA39), cuneus, and MTG] was involved. DMN is related to rumination and cognition functions, as well as the self-referential processes. The over-activation of the DMN reflects excessive self-referential thoughts, which are related with negative cognition. Chao et al. (66) proved that the dysregulation of the DMN played an important role in depression. Xu et al. (67) discovered an increased activation of DMN in depression during the resting state. As the network node of DMN, PHG is the center of the cognitive systems in emotion recognition (68). The activation of PHG is related with emotional memory encoding (69). Chen et al. (70) found a positive correlation between the activation of right PHG and depressive symptoms. The cuneus belongs to the visual areas; the activation of the sensory-related brain areas is a prerequisite to the generation of consciousness (8, 71). Yuan and Zhang et al. proved that the left cuneus exhibited a decreased activation in depressive patients (72, 73). However, task performance seems to be more in favor of the right cuneus in depression (74). The MTG is mainly responsible for facial recognition and explicit memory, which refers to a memory retrieval mode that requires the involvement of the consciousness (75). The abnormal activity of MTG may be responsible for negative emotion and cognitive impairment (76). The STG is a part of the facial-response region; the hyperactivation of the STG increased sensitivity to faces (77). Miller et al. (20) detected an overactivity centered at STG in depression during the affective processing tasks. Our findings showed that depression failed to inhibit the activation of DMN, which could be the reason for depressive tendency (78). With regards to deactivated brain areas, the abnormal network entailed salience network (SN, including IPL and insula) and executive control network (ECN, including SFG). SN takes part in the perception and integration of emotional information. The study showed that the resting-state FC alteration of SN decreased in treatment-resistant patients with depression (79). The insula is responsible for regulating attention, working memory, and some other higher cognitive functions and facilitating the processing of task-related information (80–82). In addition, the activation of the insula makes individuals prone to neuroticism, which increases sensitivity to negative stimuli (83). IPL is involved in socio-emotional processing and sensory integration (84). Lu et al. (85) found that the deactivation of the IPL was associated with anhedonia in depression. The SFG is responsible for self-consciousness and cognitive control (86). Zhao et al. (87) proved that the FC of SFG decreased in depressive patients during the resting state. Abnormal activity of the DMN, SN, and ECN together constitute a “triple network” model, which is involved in various psychiatric and neurological disorders including depression (88–90). During task performance, the ECN has a close relationship with SN; ECN usually has synergistic effects on SN (91). Meanwhile, SN can deactivate DMN (80). In the present study, we found triple-network abnormalities during emotion recognition tasks in depressive patients. Moreover, the activation of the DMN and deactivation of SN and ECN during the explicit tasks in depression are consistent with the findings of resting-state FC (67, 79, 87). In addition, when performing an explicit emotion recognition task, depressive patients are asked to classify emotional stimuli. During this process, subjects receive emotion-related information through multiple sensory input pathways, and high-level cognitive systems are engaged in processing the information and forming a perception. Haxby et al. (77) proposed three key face-selective regions when subjects perceived facial information: the occipital face area (OFA), the fusiform face area (FFA), and the superior temporal sulcus area (pSTS-FA) (92). The OFA and FFA are in charge of processing static facial information. The pSTS-FA plays a crucial role in processing dynamic features of eyes and mouth, and facilitating the identification of facial expressions (92–94). Our results showed the decreased neuro-activity of the pSTS-FA (insula and STG) in depressive patients (95), which were consistent with previous studies (20, 21). The aberrant activity of the pSTS-FA in depression indicated that depressive patients may perform poorly in processing dynamic emotional information.

To sum up, the MTG, STG, PHG, cuneus, IPL, insula, and SFG are the characteristic areas in processing emotional information consciously, and the abnormal activation of these areas could be related to poor emotional processing in patients with depression, which could be used as target brain regions for diagnosis or treatment of depression.

### 4.2. The activity changes of brain regions in response to negative/positive emotion processing

When identifying negative emotion stimuli, depression was associated with an increased activation on the precentral gyrus (BA 6), MFG (BA 6), precuneus (BA 7), and STG (BA 21). Among them, the precentral gyrus, MFG, and precuneus were attributed to DMN node. MFG is a part of the dorsolateral prefrontal cortex, which is involved in cognitive control and emotion processing (96). Zhang et al. (97) found that the MFG was responsible for aberrant negative emotion processing. The precentral gyrus is a crucial part of dorsal medial prefrontal cortex (dmPFC), which plays a critical role in the regulation of social cognition (98). Research discovered that precentral gyrus tended to process negative information, and increased activity of dmPFC was associated with impairment of processing negative emotion (17). The precuneus participates in a variety of cognitive functions including perception and memory cognition (68). Lin et al. (83) reported that the left STG was negatively correlated with neuroticism in the resting-state. Neuroticism is a personality and includes multiple dimensions. Lyon et al. (99) concluded that the dimensions of neuroticism such as anxiety, hostility and self-consciousness of neuroticism were positively related with depression. Ding et al. (100) proved that, compared to neutral stimuli, individuals exhibited a higher activation of the bilateral STG during sad stimuli. In summary, the aberrant activation of precentral gyrus, MFG, precuneus, and STG were responsible for the deficits in negative emotion processing.

As for positive emotion stimuli, the hyperactivity of the medial frontal gyrus (BA 10), MTG (BA 39), and insula (BA 13) were mainly responsible for the abnormal emotion processing, while no hypoactivation was observed. The DMN node (medial frontal gyrus), ECN node (MTG), and SN node (insula) were predominately involved. Watson and de Gelder (101) found an activation in insula, SFG, and medial frontal gyrus in explicit emotion tasks. In addition, the activation of the prefrontal cortices and insula may indicate a higher cognitive effort during identification of the positive emotion (102). Li and Wang (21) revealed a higher activation in the insula in the processing of emotion, which was consistent with our result. Medial frontal gyrus is the main region related to emotion perception (101, 103).

### 4.3. The different activity changes of brain regions between adolescents with depression and adults with depression

Compared with depressive adults, we discovered that the abnormal brain regions of depressive adolescents were more concentrated in the limbic system and striatum (caudate and lentiform nucleus). The limbic system and striatum are known as the emotional regulation system. Xia et al. (104) held that the limbic system was associated with emotion processes, and any abnormality in the limbic system could induce impairment of emotional control and render adolescents more impulsive. Tottenham et al. (105) pointed out that adverse experience could affect brain development in early life, especially the limbic structures such as the hippocampus were most sensitive to adverse experiences. In addition, accelerated or delayed limbic structure maturation might account for children's and adolescents' behavioral inadequacies which could be a risk factor for depression (106). Consistent with our results, Harlé et al. (107) found an increased activation of the striatum, particularly the caudate, in depressive adolescents. The striatum is one of the important structures in mood regulation and reward processing (108, 109). Reward is the stimuli that promotes organisms' efforts to obtain what is necessary for survival (110). The impairment in the reward system was inversely correlated with negative affection, irritability, and anhedonia in depression (107). The striatal response to reward tasks is the manifestation of depression (111, 112). Tseng et al. (113) found a positive correlation between irritability and striatal activation. The younger the age, the stronger the correlation between increased striatal activation and irritability. Anderson et al. (114) discovered that a higher functional connectivity of striatum may be responsible for anhedonia.

### 4.4. The activity changes of brain regions in patients with severe depression

The decreased activity of the SN (IPL and postcentral gyrus) and ECN (SFG) were also detected in patients with severe depression. Studies showed that discrepant severity of depression manifested differential brain activation (115, 116). Lin et al. (116) constructed prediction models for depression (mild, moderate, and severe); they found that the fMRI signals of IPL, postcentral gyrus, and SFG were able to classify the severity of depression. Liu et al. (117) detected the decreased activation of the SFG, postcentral gyrus, and IPL in patients with severe depressive. In addition, we found that the amygdala was deactivated in severe depression. However, the activation of the amygdala in our study was inconsistent with the previous task-based meta-analyses, which concluded that the overactivity of amygdala had a bearing on the impaired emotional processing in depression (21, 118). The amygdala is a limbic structure located in the medial temporal lobe, which has the ability to identify emotion (119). Moreover, the amygdala is the “behavioral brake” that protects organisms from potential harm (120). Ernst et al. (121) pointed that a weak harm-avoidant system (amygdala) could contribute to depression. Meyer et al. (122) discovered that amygdala activation was negatively associated with depressive symptoms. Lu et al. (123) recorded the impaired connectivity of amygdala during facial emotional stimulus in depressive patients. Since studies exploring abnormal brain regions in different severities of depression are rarely reported, future studies are needed to address this issue.

### 4.5. Implications for future studies

Our results showed abnormal brain regions of depression included the DMN, SN, and ECN. The coupling of between/within “triple network” during explicit tasks in depression needs further investigation. The correlation of the abnormal brain areas and depressive symptoms was unknown. Fisher's *Z*-values could be extracted for correlation analysis, or meta-regression could be performed. In the present study, we focused on the brain activity during explicit emotion tasks; further research could investigate the differential activity changes of brain regions between explicit emotion tasks and implicit emotion tasks in depression. In the future, researchers could utilize a multimodal combination with electroencephalogram, functional near-infrared spectroscopy, or single photon emission computed tomography to thoroughly investigate the neurophysiological mechanisms of depression.

### 4.6. Strengths and limitation

There were several strengths of this study. First, this is the first neuroimaging meta-analysis that explored abnormal brain activation during the performance of explicit tasks in depression. Second, we used ALE to synthesize the coordinates from included studies, which increase the credibility of the results to some extent. Third, sensitivity analyses were conducted to evaluate the robustness of the findings.

However, some limitations existed in the present study, so the results should be interpreted with caution. First, we used uncorrected *p*-value for the spatial consistency of brain activation; the uncorrected threshold may cause the possibility of false positives. Second, there was no restriction on the handedness of included patients, which could be an influencing factor to the results. Third, due to limited included studies, we failed to investigate the brain activity of different severities of depression, which needs further researches.

## 5. Conclusions

Among patients with depression, a broad range of brain areas was involved in a deficit of conscious emotion processing. The activation of brain regions was different in response to positive or negative stimuli. Due to potential clinical heterogeneity, the findings should be treated with caution.

## Data availability statement

The original contributions presented in the study are included in the article/Supplementary material, further inquiries can be directed to the corresponding authors.

## Author contributions

X-yG, Y-xL, and L-xG designed the protocol and drafted the manuscript. H-rL, JL, and R-jJ revised this manuscript. D-lZ, X-bL, H-sX, JZ, JF, and YZ screened the articles, extracted data, and conducted data synthesis. JL and S-cA originated the research question and guided the whole process of this review. All authors contributed to the article and approved the submitted version.

## References

[1] Ferrari AJ Santomauro DF Herrera AMM Shadid J Ashbaugh C Erdkine HE. Global, Regional, and National Burden of 12 Mental Disorders in 204 Countries and Territories, 1990-2019: a systematic analysis for the global burden of disease study 2019. Lancet Psychiatry. (2022) 9:137–50. 10.1016/S2215-0366(21)00395-3 35026139 PMC8776563

[2] Ettman CK Abdalla SM Cohen GH Sampson L Vivier PM Galea S. Prevalence of depression symptoms in us adults before and during the Covid-19 pandemic. JAMA Netw Open. (2020) 3:e2019686. 10.1001/jamanetworkopen.2020.19686 32876685 PMC7489837

[3] Ettman CK Cohen GH Abdalla SM Sampson L Trinquart L Castrucci BC . Persistent depressive symptoms during Covid-19: a national, population-representative, longitudinal study of US adults. Lancet Reg Health Am. (2022) 5:100091. 10.1016/j.lana.2021.100091 34635882 PMC8488314

[4] Conradi HJ Ormel J de Jonge P. Presence of individual (Residual) symptoms during depressive episodes and periods of remission: a 3-year prospective study. Psychol Med. (2011) 41:1165–74. 10.1017/S0033291710001911 20932356

[5] Han K Yang S Jia W Wang S Song Y Cao W . Health-related quality of life and its correlation with depression among Chinese centenarians. Front Public Health. (2020) 8:580757. 10.3389/fpubh.2020.580757 33194985 PMC7661682

[6] Singhal A Ross J Seminog O Hawton K Goldacre MJ. Risk of self-harm and suicide in people with specific psychiatric and physical disorders: comparisons between disorders using english national record linkage. J R Soc Med. (2014) 107:194–204. 10.1177/0141076814522033 24526464 PMC4023515

[7] Greenberg PE Fournier AA Sisitsky T Simes M Berman R Koenigsberg SH . The economic burden of adults with major depressive disorder in the United States (2010 and 2018). Pharmacoeconomics. (2021) 39:653–65. 10.1007/s40273-021-01019-4 33950419 PMC8097130

[8] Levinson M Podvalny E Baete SH He BJ. Cortical and subcortical signatures of conscious object recognition. Nat Commun. (2021) 12:2930. 10.1038/s41467-021-23266-x 34006884 PMC8131711

[9] Etkin A Büchel C Gross JJ. The neural bases of emotion regulation. Nat Rev Neurosci. (2015) 16:693–700. 10.1038/nrn4044 26481098

[10] Wagenbreth C Wattenberg L Heinze HJ Zaehle T. Implicit and explicit processing of emotional facial expressions in Parkinson's disease. Behav Brain Res. (2016) 303:182–90. 10.1016/j.bbr.2016.01.059 26850933

[11] Geurten M Salmon E Bastin C. Impaired explicit self-awareness but preserved behavioral regulation in patients with Alzheimer disease. Aging Ment Health. (2021) 25:142–8. 10.1080/13607863.2019.1675142 31599182

[12] Müller VI Cieslik EC Serbanescu I Laird AR Fox PT Eickhoff SB. Altered brain activity in unipolar depression revisited: meta-analyses of neuroimaging studies. JAMA Psychiatry. (2017) 74:47–55. 10.1001/jamapsychiatry.2016.2783 27829086 PMC5293141

[13] Krause FC Linardatos E Fresco DM Moore MT. Facial emotion recognition in major depressive disorder: a meta-analytic review. J Affect Disord. (2021) 293:320–8. 10.1016/j.jad.2021.06.053 34229285 PMC8457509

[14] Ferrer I Alacreu-Crespo A Salvador A Genty C Dubois J Sénèque M . I cannot read your eye expression: suicide attempters have difficulties in interpreting complex social emotions. Front Psychiatry. (2020) 11:543889. 10.3389/fpsyt.2020.543889 33240116 PMC7683427

[15] Wang Y Guobule N Li M Li J. The correlation of facial emotion recognition in patients with drug-naïve depression and suicide ideation. J Affect Disord. (2021) 295:250–4. 10.1016/j.jad.2021.08.051 34482056

[16] Liedtke C Kohl W Kret ME Koelkebeck K. Emotion recognition from faces with in- and out-group features in patients with depression. J Affect Disord. (2018) 227:817–23. 10.1016/j.jad.2017.11.085 29689696

[17] Li L Li R Shen F Wang X Zou T Deng C . Negative bias effects during audiovisual emotional processing in major depression disorder. Hum Brain Mapp. (2022) 43:1449–62. 10.1002/hbm.25735 34888973 PMC8837587

[18] Koch K Stegmaier S Schwarz L Erb M Reinl M Scheffler K . Neural correlates of processing emotional prosody in unipolar depression. Hum Brain Mapp. (2018) 39:3419–27. 10.1002/hbm.24185 29682814 PMC6866387

[19] Briceño EM Rapport LJ Kassel MT Bieliauskas LA Zubieta JK Weisenbach SL . Age and gender modulate the neural circuitry supporting facial emotion processing in adults with major depressive disorder. Am J Geriatr Psychiatry. (2015) 23:304–13. 10.1016/j.jagp.2014.05.007 25085721 PMC4241383

[20] Miller CH Hamilton JP Sacchet MD Gotlib IH. Meta-analysis of functional neuroimaging of major depressive disorder in youth. JAMA Psychiatry. (2015) 72:1045–53. 10.1001/jamapsychiatry.2015.1376 26332700 PMC11890701

[21] Li X Wang J. Abnormal neural activities in adults and youths with major depressive disorder during emotional processing: a meta-analysis. Brain Imaging Behav. (2021) 15:1134–54. 10.1007/s11682-020-00299-2 32710330

[22] Fusar-Poli P Placentino A Carletti F Landi P Allen P Surguladze S . Functional atlas of emotional faces processing: a voxel-based meta-analysis of 105 functional magnetic resonance imaging studies. J Psychiatry Neurosci. (2009) 34:418–32. 19949718 PMC2783433

[23] Stuhrmann A Suslow T Dannlowski U. Facial emotion processing in major depression: a systematic review of neuroimaging findings. Biol Mood Anxiety Disord. (2011) 1:10. 10.1186/2045-5380-1-10 22738433 PMC3384264

[24] Delvecchio G Fossati P Boyer P Brambilla P Falkai P Gruber O . Common and distinct neural correlates of emotional processing in bipolar disorder and major depressive disorder: a voxel-based meta-analysis of functional magnetic resonance imaging studies. Eur Neuropsychopharmacol. (2012) 22:100–13. 10.1016/j.euroneuro.2011.07.003 21820878

[25] Groenewold NA Opmeer EM de Jonge P Aleman A Costafreda SG. Emotional valence modulates brain functional abnormalities in depression: evidence from a meta-analysis of Fmri studies. Neurosci Biobehav Rev. (2013) 37:152–63. 10.1016/j.neubiorev.2012.11.015 23206667

[26] Lai C-H. Patterns of cortico-limbic activations during visual processing of sad faces in depression patients: a coordinate-based meta-analysis. J Neuropsychiatry Clin Neurosci. (2014) 26:34–43. 10.1176/appi.neuropsych.12060143 24275771

[27] Zhang Z Huang P Li S Liu Z Zhang J Li Y . Neural mechanisms underlying the processing of emotional stimuli in individuals with depression: an ale meta-analysis study. Psychiatry Res. (2022) 313:114598. 10.1016/j.psychres.2022.114598 35544984

[28] Critchley H Daly E Phillips M Brammer M Bullmore E Williams S . Explicit and implicit neural mechanisms for processing of social information from facial expressions: a functional magnetic resonance imaging study. Hum Brain Mapp. (2000) 9:93–105. 10.1002/(SICI)1097-0193(200002)9:2<93::AID-HBM4>3.0.CO;2-Z 10680766 PMC6872127

[29] Marrazzo G Vaessen MJ de Gelder B. Decoding the difference between explicit and implicit body expression representation in high level visual, prefrontal and inferior parietal cortex. Neuroimage. (2021) 243:118545. 10.1016/j.neuroimage.2021.118545 34478822

[30] Pierce JE Thomasson M Voruz P Selosse G Péron J. Explicit and implicit emotion processing in the cerebellum: a meta-analysis and systematic review. Cerebellum. (2022). 10.1007/s12311-022-01459-4 35999332 PMC10485090

[31] Page MJ McKenzie JE Bossuyt PM Boutron I Hoffmann TC Mulrow CD . The prisma 2020 statement: an updated guideline for reporting systematic reviews. BMJ (Clin Res ed). (2021) 372:n71. 10.1136/bmj.n71 33782057 PMC8005924

[32] Shimizu K Kikuchi S Kobayashi T Kato S. Persistent complex bereavement disorder: clinical utility and classification of the category proposed for diagnostic and statistical manual of mental disorders, 5th Edition. Psychogeriatrics. (2017) 17:17–24. 10.1111/psyg.12183 26781759

[33] Implementation Implementation of the international statistical classification of diseases and related health problems tenth revision (Icd-10). Epidemiol Bull. (1997) 18:1–4. 9197082

[34] Chen YF. Chinese classification of mental disorders (Ccmd-3): towards integration in international classification. Psychopathology. (2002) 35:171–5. 10.1159/000065140 12145505

[35] Stang A. Critical evaluation of the Newcastle-Ottawa scale for the assessment of the quality of nonrandomized studies in meta-analyses. Eur J Epidemiol. (2010) 25:603–5. 10.1007/s10654-010-9491-z 20652370

[36] Gentili C Messerotti Benvenuti S Lettieri G Costa C Cecchetti L. Roi and Phobias: the effect of roi approach on an ale meta-analysis of specific phobias. Hum Brain Mapp. (2019) 40:1814–28. 10.1002/hbm.24492 30548734 PMC6865604

[37] Li ZL. Fmri study of emotional face stimulation in normal subjects and depression (Master). Kunming Medical University (2007).

[38] Yao ZJ. Functional magnetic resonance imaging of brain and its clinical application in major depression (Doctor). Southeast University (2008).

[39] Cao Y Yao Z Xie S. Neural substrates for explicit recognition of dynamic facial expressions in male patients with major depressive disorder: a Fmri study. Chin Mental Health J. (2008) 22:265–70. 10.3321/j.issn:1000-6729.2008.04.00730704229

[40] Yang TT Simmons AN Matthews SC Tapert SF Frank GK Max JE . Adolescents with major depression demonstrate increased amygdala activation. J Am Acad Child Adolesc Psychiatry. (2010) 49:42–51. 10.1097/00004583-201001000-00008 20215925 PMC2935523

[41] Townsend JD Eberhart NK Bookheimer SY Eisenberger NI Foland-Ross LC Cook IA . Fmri activation in the amygdala and the orbitofrontal cortex in unmedicated subjects with major depressive disorder. Psychiatry Res. (2010) 183:209–17. 10.1016/j.pscychresns.2010.06.001 20708906 PMC3382985

[42] Scheuerecker J Meisenzahl EM Koutsouleris N Roesner M Schöpf V Linn J . Orbitofrontal volume reductions during emotion recognition in patients with major depression. J Psychiatry Neurosci. (2010) 35:311–20. 10.1503/jpn.090076 20569645 PMC2928284

[43] Derntl B Seidel E-M Eickhoff SB Kellermann T Gur RC Schneider F . Neural correlates of social approach and withdrawal in patients with major depression. Soc Neurosci. (2011) 6:482–501. 10.1080/17470919.2011.579800 21777105 PMC3203307

[44] van Wingen GA van Eijndhoven P Tendolkar I Buitelaar J Verkes RJ Fernández G. Neural basis of emotion recognition deficits in first-episode major depression. Psychol Med. (2011) 41:1397–405. 10.1017/S0033291710002084 21054920

[45] Ritchey M Dolcos F Eddington KM Strauman TJ Cabeza R. Neural correlates of emotional processing in depression: changes with cognitive behavioral therapy and predictors of treatment response. J Psychiatr Res. (2011) 45:577–87. 10.1016/j.jpsychires.2010.09.007 20934190 PMC3042483

[46] Schlund MW Verduzco G Cataldo MF Hoehn-Saric R. Generalized anxiety modulates frontal and limbic activation in major depression. Behav Brain Funct. (2012) 8:8. 10.1186/1744-9081-8-8 22321875 PMC3293052

[47] Zhong M Yao S Zhu X Yi J Zhu X Wang X . Elevated amygdala activity to negative faces in young adults with early onset major depressive disorder. Psychiatry Research-Neuroimaging. (2012) 201:107–12. 10.1016/j.pscychresns.2011.06.003 22398297

[48] Tu J. The effects of emotional processing on cognitive control in depression: an Fmri study (Master). Third Military Medical University (2012).

[49] Li J Xu C Cao X Gao Q Wang Y Wang Y . Abnormal activation of the occipital lobes during emotion picture processing in major depressive disorder patients (star star star) star. Neural Regen Res. (2013) 8:1693–701. 10.4103/1673-5374.121696 25206466 PMC4145913

[50] Lisiecka DM Carballedo A Fagan AJ Ferguson Y Meaney J Frodl T. Recruitment of the left hemispheric emotional attention neural network in risk for and protection from depression. J Psychiatry Neurosci. (2013) 38:117–28. 10.1503/jpn.110188 23010257 PMC3581592

[51] Bian HM. Fmri study of negative emotional image stimulation in female patients with first episode major depression (Master). Tianjin Medical University (2013).25864196

[52] Skokauskas N Carballedo A Fagan A Frodl T. The role of sexual abuse on functional neuroimaging markers associated with major depressive disorder. World J Biol Psychiatry. (2015) 16:513–20. 10.3109/15622975.2015.1048723 26114449

[53] Murrough JW Collins KA Fields J DeWilde KE Phillips ML Mathew SJ . Regulation of neural responses to emotion perception by ketamine in individuals with treatment-resistant major depressive disorder. Transl Psychiatry. (2015) 5:e509. 10.1038/tp.2015.10 25689570 PMC4445748

[54] Cai Y Li W Li Z Zhang L Liao M Liu B . The characteristics of brain function activity between patients with bipolar depression and major depressive disorder: a functional magnetic resonance imaging study of affective pictures task. Chin J Psychiatry. (2016) 49:202–9. 10.3760/cma.j.issn.1006-7884.2016.04.00330704229

[55] Ho TC Zhang S Sacchet MD Weng H Connolly CG Henje Blom E . Fusiform gyrus dysfunction is associated with perceptual processing efficiency to emotional faces in adolescent depression: a model-based approach. Front Psychol. (2016) 7:40. 10.3389/fpsyg.2016.00040 26869950 PMC4740953

[56] Xu ZX. Brain functional imaging study of facial dynamic emotion recognition in patients with major depression (Doctor). Beijing University of Chinese Medicine (2017).

[57] Bürger C Redlich R Grotegerd D Meinert S Dohm K Schneider I . Differential abnormal pattern of anterior cingulate gyrus activation in unipolar and bipolar depression: an Fmri and pattern classification approach. Neuropsychopharmacology. (2017) 42:1399–408. 10.1038/npp.2017.36 28205606 PMC5436122

[58] Mel'nikov ME Petrovskii ED Bezmaternykh DD Kozlova LI Shtark MB Savelov AA . Fmri responses in healthy individuals and in patients with mild depression to presentation of pleasant and unpleasant images. Bull Exp Biol Med. (2018) 164:601–4. 10.1007/s10517-018-4040-y 29577204

[59] Groves SJ Pitcher TL Melzer TR Jordan J Carter JD Malhi GS . Brain activation during processing of genuine facial emotion in depression: preliminary findings. J Affect Disord. (2018) 225:91–6. 10.1016/j.jad.2017.07.049 28802727

[60] Song X Mu X Yu M. The study of brain Fmri in task state of first-episode patients with mild and moderate depression before and after treatment. J Clin Radiol. (2019) 38:1174–9.

[61] Nagy SA Kürtös Z Németh N Perlaki G Csernela E Lakner FE . Childhood maltreatment results in altered deactivation of reward processing circuits in depressed patients: a functional magnetic resonance imaging study of a facial emotion recognition task. Neurobiol Stress. (2021) 15:100399. 10.1016/j.ynstr.2021.100399 34646916 PMC8495173

[62] Lui S Zhou XJ Sweeney JA Gong Q. Psychoradiology: the Frontier of neuroimaging in psychiatry. Radiology. (2016) 281:357–72. 10.1148/radiol.2016152149 27755933 PMC5084981

[63] Lai H Kong X Zhao Y Pan N Zhang X He M . Patterns of a structural covariance network associated with dispositional optimism during late adolescence. Neuroimage. (2022) 251:119009. 10.1016/j.neuroimage.2022.119009 35182752

[64] Suo X Zuo C Lan H Pan N Zhang X Kemp GJ . Covid-19 vicarious traumatization links functional connectome to general distress. Neuroimage. (2022) 255:119185. 10.1016/j.neuroimage.2022.119185 35398284 PMC8986542

[65] Li F Sun H Biswal BB Sweeney JA Gong Q. Artificial intelligence applications in psychoradiology. Psychoradiology. (2021) 1:94–107. 10.1093/psyrad/kkab009 PMC10594695 37881257

[66] Chao ZC Dillon DG Liu YH Barrick EM Wu CT. Altered coordination between frontal delta and parietal alpha networks underlies anhedonia and depressive rumination in major depressive disorder. J Psychiatry Neurosci. (2022) 47:E367–e78. 10.1503/jpn.220046 36318983 PMC9633055

[67] Xu Z Zhao W Wang H Tian Y Lei X. Functional connectivity between dorsal attention and default mode networks mediates subjective sleep duration and depression in young females. J Affect Disord. (2023) 325:386–91. 10.1016/j.jad.2023.01.023 36634855

[68] Morales J Lau H Fleming SM. Domain-general and domain-specific patterns of activity supporting metacognition in human prefrontal cortex. J Neurosci. (2018) 38:3534–46. 10.1523/JNEUROSCI.2360-17.2018 29519851 PMC5895040

[69] Aminoff EM Kveraga K Bar M. The role of the parahippocampal cortex in cognition. Trends Cogn Sci. (2013) 17:379–90. 10.1016/j.tics.2013.06.009 23850264 PMC3786097

[70] Chen XF He P Xu KH Jin YH Chen Y Wang B . Disrupted spontaneous neural activity and its interaction with pain and emotion in temporomandibular disorders. Front Neurosci. (2022) 16:941244. 10.3389/fnins.2022.941244 36090263 PMC9453298

[71] Ahrweiler N Santana-Gonzalez C Zhang N Quandt G Ashtiani N Liu G . Neural activity associated with symptoms change in depressed adolescents following self-processing neurofeedback. Brain Sci. (2022) 12:1128. 10.3390/brainsci12091128 36138864 PMC9496932

[72] Yuan J Yu H Yu M Liang X Huang C He R . Altered spontaneous brain activity in major depressive disorder: an activation likelihood estimation meta-analysis. J Affect Disord. (2022) 314:19–26. 10.1016/j.jad.2022.06.014 35750093

[73] Zhang X Zhang R Lv L Qi X Shi J Xie S. Correlation between cognitive deficits and dorsolateral prefrontal cortex functional connectivity in first-episode depression. J Affect Disord. (2022) 312:152–8. 10.1016/j.jad.2022.06.024 35752217

[74] Zhang WN Chang SH Guo LY Zhang KL Wang J. The neural correlates of reward-related processing in major depressive disorder: a meta-analysis of functional magnetic resonance imaging studies. J Affect Disord. (2013) 151:531–9. 10.1016/j.jad.2013.06.039 23856280

[75] Xu J Lyu H Li T Xu Z Fu X Jia F . Delineating functional segregations of the human middle temporal gyrus with resting-state functional connectivity and coactivation patterns. Hum Brain Mapp. (2019) 40:5159–71. 10.1002/hbm.24763 31423713 PMC6865466

[76] Zhang Q Li X Yan H Wang Y Ou Y Yu Y . Associations between abnormal spontaneous neural activity and clinical variables, eye movements, and event-related potential indicators in major depressive disorder. Front Neurosci. (2022) 16:1056868. 10.3389/fnins.2022.1056868 36711124 PMC9875062

[77] Haxby JV Hoffman EA Gobbini MI. The distributed human neural system for face perception. Trends Cogn Sci. (2000) 4:223–33. 10.1016/S1364-6613(00)01482-0 10827445

[78] Han DH Kim SM Bae S Renshaw PF Anderson JS. A failure of suppression within the default mode network in depressed adolescents with compulsive internet game play. J Affect Disord. (2016) 194:57–64. 10.1016/j.jad.2016.01.013 26802508

[79] Sun J Ma Y Guo C Du Z Chen L Wang Z . Distinct patterns of functional brain network integration between treatment-resistant depression and non treatment-resistant depression: a resting-state functional magnetic resonance imaging study. Prog Neuropsychopharmacol Biol Psychiatry. (2023) 120:110621. 10.1016/j.pnpbp.2022.110621 36031163

[80] Nomi JS Farrant K Damaraju E Rachakonda S Calhoun VD Uddin LQ. Dynamic functional network connectivity reveals unique and overlapping profiles of insula subdivisions. Hum Brain Mapp. (2016) 37:1770–87. 10.1002/hbm.23135 26880689 PMC4837017

[81] Lamm C Singer T. The role of anterior insular cortex in social emotions. Brain Struct Funct. (2010) 214:579–91. 10.1007/s00429-010-0251-3 20428887

[82] Kim SH An K Namkung H Saito A Rannals MD Moore JR . Anterior insula-associated social novelty recognition: pivotal roles of a local retinoic acid cascade and oxytocin signaling. Am J Psychiatry. (2022) 180:305–17. 10.1101/2021.01.15.426848 36128683 PMC13520127

[83] Lin J Li L Pan N Liu X Zhang X Suo X . Neural correlates of neuroticism: a coordinate-based meta-analysis of resting-state functional brain imaging studies. Neurosci Biobehav Rev. (2023) 146:105055. 10.1016/j.neubiorev.2023.105055 36681370

[84] Camacho MC Karim HT Perlman SB. Neural architecture supporting active emotion processing in children: a multivariate approach. Neuroimage. (2019) 188:171–80. 10.1016/j.neuroimage.2018.12.013 30537564 PMC6401267

[85] Lu S Shao J Feng Q Wu C Fang Z Jia L . Aberrant interhemispheric functional connectivity in major depressive disorder with and without anhedonia. BMC Psychiatry. (2022) 22:688. 10.1186/s12888-022-04343-x 36348342 PMC9644581

[86] du Boisgueheneuc F Levy R Volle E Seassau M Duffau H Kinkingnehun S . Functions of the left superior frontal gyrus in humans: a lesion study. Brain. (2006) 129:3315–28. 10.1093/brain/awl244 16984899

[87] Zhao Q Swati ZNK Metmer H Sang X Lu J. Investigating executive control network and default mode network dysfunction in major depressive disorder. Neurosci Lett. (2019) 701:154–61. 10.1016/j.neulet.2019.02.045 30831152

[88] Menon V. Large-scale brain networks and psychopathology: a unifying triple network model. Trends Cogn Sci. (2011) 15:483–506. 10.1016/j.tics.2011.08.003 21908230

[89] Liang X He Y Salmeron BJ Gu H Stein EA Yang Y. Interactions between the salience and default-mode networks are disrupted in cocaine addiction. J Neurosci. (2015) 35:8081–90. 10.1523/JNEUROSCI.3188-14.2015 26019326 PMC4444534

[90] Li Q Liu J Wang W Wang Y Li W Chen J . Disrupted coupling of large-scale networks is associated with relapse behaviour in heroin-dependent men. J Psychiatry Neurosci. (2018) 43:48–57. 10.1503/jpn.170011 29252165 PMC5747535

[91] Bolton TAW Wotruba D Buechler R Theodoridou A Michels L Kollias S . Triple network model dynamically revisited: lower salience network state switching in pre-psychosis. Front Physiol. (2020) 11:66. 10.3389/fphys.2020.00066 32116776 PMC7027374

[92] Grill-Spector K Weiner KS Kay K Gomez J. The functional neuroanatomy of human face perception. Annu Rev Vis Sci. (2017) 3:167–96. 10.1146/annurev-vision-102016-061214 28715955 PMC6345578

[93] Deen B Saxe R. Parts-based representations of perceived face movements in the superior temporal sulcus. Hum Brain Mapp. (2019) 40:2499–510. 10.1002/hbm.24540 30761664 PMC6865455

[94] Bernstein M Erez Y Blank I Yovel G. An integrated neural framework for dynamic and static face processing. Sci Rep. (2018) 8:7036. 10.1038/s41598-018-25405-9 29728577 PMC5935689

[95] Zhen Z Fang H Liu J. The hierarchical brain network for face recognition. PLoS ONE. (2013) 8:e59886. 10.1371/journal.pone.0059886 23527282 PMC3603994

[96] Dumitru A Rocchi L Saini F Rothwell JC Roiser JP David AS . Influence of theta-burst transcranial magnetic stimulation over the dorsolateral prefrontal cortex on emotion processing in healthy volunteers. Cogn Affect Behav Neurosci. (2020) 20:1278–93. 10.3758/s13415-020-00834-0 33000366 PMC7716858

[97] Zhang S Zhang Y Ma W Qi Z Wang Y Tao Q. Neural correlates of negative emotion processing in subthreshold depression. Soc Cogn Affect Neurosci. (2022) 17:655–61. 10.1093/scan/nsac003 35156124 PMC9250298

[98] Sadeghi S Schmidt SNL Mier D Hass J. Effective connectivity of the human mirror neuron system during social cognition. Soc Cogn Affect Neurosci. (2022) 17:732–43. 10.1093/scan/nsab138 35086135 PMC9340111

[99] Lyon KA Elliott R Ware K Juhasz G Brown LJ. Associations between facets and aspects of big five personality and affective disorders: a systematic review and best evidence synthesis. J Affect Disord. (2021) 288:175–88. 10.1016/j.jad.2021.03.061 33901698

[100] Ding J Wang Y Wang C d'Oleire Uquillas F He Q Cheng L . Negative impact of sadness on response inhibition in females: an explicit emotional stop signal task Fmri study. Front Behav Neurosci. (2020) 14:119. 10.3389/fnbeh.2020.00119 32903296 PMC7396530

[101] Watson R de Gelder B. How white and black bodies are perceived depends on what emotion is expressed. Sci Rep. (2017) 7:41349. 10.1038/srep41349 28128279 PMC5269713

[102] Disner SG Beevers CG Haigh EA Beck AT. Neural mechanisms of the cognitive model of depression. Nat Rev Neurosci. (2011) 12:467–77. 10.1038/nrn3027 21731066

[103] Bae S Kang KD Kim SW Shin YJ Nam JJ Han DH. Investigation of an emotion perception test using functional magnetic resonance imaging. Comput Methods Programs Biomed. (2019) 179:104994. 10.1016/j.cmpb.2019.104994 31443867

[104] Xia Y Zhuang K Sun J Chen Q Wei D Yang W . Emotion-related brain structures associated with trait creativity in middle children. Neurosci Lett. (2017) 658:182–8. 10.1016/j.neulet.2017.08.008 28780167

[105] Tottenham N Sheridan MA. A review of adversity, the amygdala and the hippocampus: a consideration of developmental timing. Front Hum Neurosci. (2009) 3:68. 10.3389/neuro.09.068.2009 20161700 PMC2813726

[106] Albar Z Sattar A. Effects of parental internalizing and externalizing behavior problems on children's limbic brain structures-an Mri study. Brain Sci. (2022) 12:1319. 10.3390/brainsci12101319 36291253 PMC9599765

[107] Harlé KM Ho TC Connolly CG Simmons A Yang TT. How obstructed action efficacy impacts reward-based decision making in adolescent depression: an Fmri study. J Am Acad Child Adolesc Psychiatry. (2023) 20:S0890-8567(23)00130-2. 10.1016/j.jaac.2023.01.024 36948392

[108] Lu F Cui Q He Z Sheng W Pang Y Chen Y . Prefrontal-limbic-striatum dysconnectivity associated with negative emotional endophenotypes in bipolar disorder during depressive episodes. J Affect Disord. (2021) 295:422–30. 10.1016/j.jad.2021.08.055 34507222

[109] Di Martino A Scheres A Margulies DS Kelly AM Uddin LQ Shehzad Z . Functional connectivity of human striatum: a resting state fmri study. Cereb Cortex. (2008) 18:2735–47. 10.1093/cercor/bhn041 18400794

[110] Schultz W. Dopamine reward prediction-error signalling: a two-component response. Nat Rev Neurosci. (2016) 17:183–95. 10.1038/nrn.2015.26 26865020 PMC5549862

[111] O'Callaghan G Stringaris A. Reward processing in adolescent depression across neuroimaging modalities. Z Kinder Jugendpsychiatr Psychother. (2019) 47:535–41. 10.1024/1422-4917/a000663 30957688 PMC6996129

[112] Rengasamy M Nance M Eckstrand K Forbes E. Splitting the reward: differences in inflammatory marker associations with neural connectivity between reward anticipation and reward outcome in adolescents at high risk for depression. J Affect Disord. (2023) 327:128–36. 10.1016/j.jad.2023.01.118 36736795 PMC11466213

[113] Tseng WL Deveney CM Stoddard J Kircanski K Frackman AE Yi JY . Brain mechanisms of attention orienting following frustration: associations with irritability and age in youths. Am J Psychiatry. (2019) 176:67–76. 10.1176/appi.ajp.2018.18040491 30336704 PMC6408218

[114] Anderson Z Damme KSF Carroll AL Ka-Yi Chat I Young KS Craske MG . Association between reward-related functional connectivity and tri-level mood and anxiety symptoms. Neuroimage Clin. (2023) 37:103335. 10.1016/j.nicl.2023.103335 36736199 PMC9926301

[115] Chen F Wang L Ding Z. Alteration of whole-brain amplitude of low-frequency fluctuation and degree centrality in patients with mild to moderate depression: a resting-state functional magnetic resonance imaging study. Front Psychiatry. (2022) 13:1061359. 10.3389/fpsyt.2022.1061359 36569607 PMC9768018

[116] Lin C Lee SH Huang CM Chen GY Chang W Liu HL . Automatic diagnosis of late-life depression by 3d convolutional neural networks and cross-sample entropy analysis from resting-state Fmri. Brain Imaging Behav. (2023) 17:125–35. 10.1007/s11682-022-00748-0 36418676 PMC9922223

[117] Liu X Wang Y Liu H Liu Z Zhou W. Diffusion tensor imaging and resting state functional magnetic resonance imaging on young patients with major depressive disorder. J Cent South Univ (Med Sci). (2010) 35:25–31. 10.3969/j.issn.1672-7347.2010.01.004 20130361

[118] Liu Y Liu Y Lin W He Z Zhang D Guan Q . The core neural mechanisms underlying depression disorder: a meta-analysis of Fmri studies. Scientia Sinica Vitae. (2015) 45:1214–23. 10.1360/N052015-00164

[119] Bujarski KA Song Y Xie T Leeds Z Kolankiewicz SI Wozniak GH . Modulation of emotion perception via amygdala stimulation in humans. Front Neurosci. (2021) 15:795318. 10.3389/fnins.2021.795318 35221888 PMC8864965

[120] Zald DH. The human amygdala and the emotional evaluation of sensory stimuli. Brain Res Brain Res Rev. (2003) 41:88–123. 10.1016/S0165-0173(02)00248-5 12505650

[121] Ernst M Pine DS Hardin M. Triadic model of the neurobiology of motivated behavior in adolescence. Psychol Med. (2006) 36:299–312. 10.1017/S0033291705005891 16472412 PMC2733162

[122] Meyer K Hindi Attar C Fiebig J Stamm T Bassett TR Bauer M . Daring to feel: emotion-focused psychotherapy increases amygdala activation and connectivity in euthymic bipolar disorder. A randomized controlled trial. Biol Psychiatry Cogn Neurosci Neuroimaging. (2023) 8:S2451-9022(23)00046-0. 10.1016/j.bpsc.2023.02.008 36898634

[123] Lu Q Li H Luo G Wang Y Tang H Han L . Impaired prefrontal-amygdala effective connectivity is responsible for the dysfunction of emotion process in major depressive disorder: a dynamic causal modeling study on Meg. Neurosci Lett. (2012) 523:125–30. 10.1016/j.neulet.2012.06.058 22750155

