# Supplementary material for: The conscious processing of emotion in depression disorder: a meta-analysis of neuroimaging studies
Source: Front Psychiatry. 2023 Jun 28;14:1099426. doi: 10.3389/fpsyt.2023.1099426 (PMC10338122; doi:10.3389/fpsyt.2023.1099426)
Supplement: Supplementary file 1 [file Table_1.docx]

***Supplementary Material***

Table 1. search strategy

| **Search strategy of PubMed: 2363** |
| --- |
| 1.Depression/ |
| 2.Depressive Disorder/ |
| 3.depressive disorder, major/ |
| 4.Mood Disorders/ |
| 5.Depressive Disorder, Treatment-Resistant |
| 6. depress*[Title/Abstract] OR mood?[Title/Abstract] OR affective disorder*[Title/Abstract] OR affective symptom*[Title/Abstract] OR depress* disorder*[Title/Abstract] OR dysphoria[Title/Abstract] OR endogenous depression[Title/Abstract] OR involutional depression[Title/Abstract] OR major depression[Title/Abstract] OR masked depression[Title/Abstract] OR melancholia[Title/Abstract] OR mood disorder[Title/Abstract] OR organic depression[Title/Abstract] OR recurrent brief depression[Title/Abstract] OR anaclitic depression[Title/Abstract] OR recurrent depression[Title/Abstract] OR treatment resistant depression[Title/Abstract] OR dysthymic disorder[Title/Abstract] OR reactive depression[Title/Abstract] OR agitated depression[Title/Abstract] OR atypical depression[Title/Abstract] OR sadness[Title/Abstract] |
| 7.#1 OR #2 OR #3 OR #4 OR #5 |
| 8.face[Title/Abstract] OR emotion[Title/Abstract] OR Angry Face[Title/Abstract] OR Emotion Recognition[Title/Abstract] OR Facial Expression of Emotion[Title/Abstract] OR Facial Expression Perception[Title/Abstract] OR Fear Face[Title/Abstract] OR Happy Face[Title/Abstract] OR Sad Face[Title/Abstract] OR Threatening Face[Title/Abstract] OR Social Cognition[Title/Abstract] OR Fear Face Conditioning[Title/Abstract] |
| 9.magnetic resonance imaging* OR neuroimaging* OR functional magnetic resonance imaging* OR structural magnetic resonance imaging* OR BOLD OR Blood Oxygen Level-Dependent OR Positron Emission Tomography |
| 10. #7 AND #8 AND #9 |
| **Search strategy of WOS: 6586** |
| 1.TS=(Depression OR Depressive Disorder, major OR Mood Disorders OR depress* or mood? or affective disorder* or affective symptom* or depress* disorder* or dysphoria or endogenous depression or involutional depression or major depression or masked depression or melancholia or mood disorder or organic depression or recurrent brief depression or anaclitic depression or recurrent depression or treatment resistant depression or dysthymic disorder or reactive depression or agitated depression or atypical depression or sadness) |
| 2.TS=(face OR emotion OR Angry Face OR Emotion Recognition OR Facial Expression of Emotion OR Facial Expression Perception OR Fear Face OR Happy Face OR Sad Face OR Threatening Face OR Social Cognition OR Fear Face Conditioning) |
| 3.TS=(magnetic resonance imaging* OR neuroimaging* OR functional magnetic resonance imaging* OR structural magnetic resonance imaging* OR BOLD OR Blood Oxygen Level-Dependent OR Positron Emission Tomography) |
| 4. #1 AND #2 AND #3 |
| **Search strategy of Embase:722** |
| #1 'face'/exp OR face OR 'emotion'/exp OR emotion OR 'angry face' OR (angry AND ('face'/exp OR face)) OR 'emotion recognition' OR (('emotion'/exp OR emotion) AND ('recognition'/exp OR recognition)) OR 'facial expression of emotion' OR (facial AND expression AND of AND ('emotion'/exp OR emotion)) OR 'facial expression perception' OR (facial AND expression AND ('perception'/exp OR perception)) OR 'fear face' OR (('fear'/exp OR fear) AND ('face'/exp OR face)) OR 'happy face' OR (happy AND ('face'/exp OR face)) OR 'sad face' OR (sad AND ('face'/exp OR face)) OR 'threatening face' OR (threatening AND ('face'/exp OR face)) OR 'social cognition'/exp OR 'social cognition' OR (social AND ('cognition'/exp OR cognition)) OR 'fear face conditioning' OR (('fear'/exp OR fear) AND ('face'/exp OR face) AND ('conditioning'/exp OR conditioning)) |
| #2 ((((magnetic:ti,ab,kw AND resonance:ti,ab,kw AND imaging*:ti,ab,kw OR neuroimaging*:ti,ab,kw OR functional:ti,ab,kw) AND magnetic:ti,ab,kw AND resonance:ti,ab,kw AND imaging*:ti,ab,kw OR structural:ti,ab,kw) AND magnetic:ti,ab,kw AND resonance:ti,ab,kw AND imaging*:ti,ab,kw OR bold:ti,ab,kw OR blood:ti,ab,kw) AND oxygen:ti,ab,kw AND 'level dependent':ti,ab,kw OR positron:ti,ab,kw) AND emission:ti,ab,kw AND tomography:ti,ab,kw |
| #3 'depression'/exp |
| #4 (((((((((((((((((((depression:ti,ab,kw OR depressive:ti,ab,kw) AND disorder,:ti,ab,kw AND major:ti,ab,kw OR mood:ti,ab,kw) AND disorders:ti,ab,kw OR depress*:ti,ab,kw OR mood?:ti,ab,kw OR affective:ti,ab,kw) AND disorder*:ti,ab,kw OR affective:ti,ab,kw) AND symptom*:ti,ab,kw OR depress*:ti,ab,kw) AND disorder*:ti,ab,kw OR dysphoria:ti,ab,kw OR endogenous:ti,ab,kw) AND depression:ti,ab,kw OR involutional:ti,ab,kw) AND depression:ti,ab,kw OR major:ti,ab,kw) AND depression:ti,ab,kw OR masked:ti,ab,kw) AND depression:ti,ab,kw OR melancholia:ti,ab,kw OR mood:ti,ab,kw) AND disorder:ti,ab,kw OR organic:ti,ab,kw) AND depression:ti,ab,kw OR recurrent:ti,ab,kw) AND brief:ti,ab,kw AND depression:ti,ab,kw OR anaclitic:ti,ab,kw) AND depression:ti,ab,kw OR recurrent:ti,ab,kw) AND depression:ti,ab,kw OR treatment:ti,ab,kw) AND resistant:ti,ab,kw AND depression:ti,ab,kw OR dysthymic:ti,ab,kw) AND disorder:ti,ab,kw OR reactive:ti,ab,kw) AND depression:ti,ab,kw OR agitated:ti,ab,kw) AND depression:ti,ab,kw OR atypical:ti,ab,kw) AND depression:ti,ab,kw OR sadness:ti,ab,kw |
| #5 #3 OR #4 |
| #6 #1 AND #2 AND #5 |
| **Search strategy of Cochrane:1105** |
| #1 depressions [MeSH Terms] |
| #2 (Depression OR Depressive Disorder, major OR Mood Disorders OR depress* or mood? or affective disorder* or affective symptom* or depress* disorder* or dysphoria or endogenous depression or involutional depression or major depression or masked depression or melancholia or mood disorder or organic depression or recurrent brief depression or anaclitic depression or recurrent depression or treatment resistant depression or dysthymic disorder or reactive depression or agitated depression or atypical depression or sadness ):ti,ab,kw |
| #3 (face OR emotion OR Angry Face OR Emotion Recognition OR Facial Expression of Emotion OR Facial Expression Perception OR Fear Face OR Happy Face OR Sad Face OR Threatening Face OR Social Cognition OR Fear Face Conditioning):ti,ab,kw |
| #4 (magnetic resonance imaging* OR neuroimaging* OR functional magnetic resonance imaging* OR structural magnetic resonance imaging* OR BOLD OR Blood Oxygen Level-Dependent OR Positron Emission Tomography):ti,ab,kw |
| #5 #1 or #2 |
| #6 #5 AND #3 AND #4 |
| **Search strategy of CNKI: 95** |
| TKA = '核磁 OR MRI OR 神经成像 OR 功能核磁共振成像 OR PET OR BOLD OR 血氧水平依赖 OR 正电子发射断层扫描' AND TKA = '抑郁 OR 重度抑郁 OR MDD' AND TKA = '情绪识别 OR 愤怒处理 OR 快乐处理 OR 悲伤处理 OR 中性处理 OR 社会认知 OR 中性情绪 OR 负性情绪 OR 正性情绪 OR 面部表情 OR 面部表情知觉 OR 情绪图片' |
| **Search strategy of Wanfang: 387** |
| 摘要:(情绪识别 OR 愤怒处理 OR 快乐处理 OR 悲伤处理 OR 中性处理 OR 社会认知 OR 中性情绪 OR 负性情绪 OR 正性情绪 OR 面部表情 OR 面部表情知觉 OR 情绪图片) AND 主题:(抑郁 OR 重度抑郁 OR MDD) AND 摘要:(核磁 OR MRI OR 神经成像 OR 功能核磁共振成像 OR PET OR BOLD OR 血氧水平依赖 OR 正电子发射断层扫描) |
| **Search strategy of CBM:55** |
| #1 ( "情绪识别"[常用字段:智能] OR "愤怒处理"[常用字段:智能] OR "快乐处理"[常用字段:智能] OR "悲伤处理"[常用字段:智能] OR "中性处理"[常用字段:智能] OR "社会认知"[常用字段:智能] OR "中性情绪"[常用字段:智能] OR "负性情绪"[常用字段:智能] OR "正性情绪"[常用字段:智能] OR "面部表情"[常用字段:智能] OR "面部表情知觉"[常用字段:智能] OR "情绪图片"[常用字段:智能]) |
| #2 ( "抑郁"[常用字段:智能] OR "重度抑郁"[常用字段:智能] OR "MDD"[常用字段:智能]) |
| #3 ( "核磁"[常用字段:智能] OR "MRI"[常用字段:智能] OR "神经成像"[常用字段:智能] OR "功能核磁共振成像"[常用字段:智能] OR "PET"[常用字段:智能] OR "BOLD"[常用字段:智能] OR "血氧水平依赖"[常用字段:智能] OR "正电子发射断层扫描"[常用字段:智能]) |
| #4 #1 AND #2 AND #3 |
| **Search strategy of VIP:52** |
| ((M=情绪识别 OR 愤怒处理 OR 快乐处理 OR 悲伤处理 OR 中性处理 OR 社会认知 OR 中性情绪 OR 负性情绪 OR 正性情绪 OR 面部表情 OR 面部表情知觉 OR 情绪图片) OR (R=情绪识别 OR 愤怒处理 OR 快乐处理 OR 悲伤处理 OR 中性处理 OR 社会认知 OR 中性情绪 OR 负性情绪 OR 正性情绪 OR 面部表情 OR 面部表情知觉 OR 情绪图片)) AND ((M=抑郁 OR 重度抑郁 OR MDD) OR (R=抑郁 OR 重度抑郁 OR MDD)) AND ((M=核磁 OR MRI OR 神经成像 OR 功能核磁共振成像 OR PET OR BOLD OR 血氧水平依赖 OR 正电子发射断层扫描) OR (R=核磁 OR MRI OR 神经成像 OR 功能核磁共振成像 OR PET OR BOLD OR 血氧水平依赖 OR 正电子发射断层扫描)) |

**Table 2. The scoring rules of risk of bias**

|  | **Item** |  |
| --- | --- | --- |
| **Study selection** | | |
| Adequate case definition | 1 | (+): individuals with depression were defined through a clinical interview (e.g., DSM, ICD, CCMD) |
|  |  | (-): patients definition was inadequate (e.g., questionnaire cut-off, self-report or self-diagnosis) |
|  |  | (?): no or incomplete information were provided on the diagnostic process |
| Representativeness of the cases | 2 | (+): a defined procedure for inclusion was reported (recruitment center, recruitment announcement or consecutive patients screened) |
|  |  | (-): a specific selection biased was introduced |
|  |  | (?): no information was presented |
| Selection of Controls | 3 | (+): healthy controls were chosen from the same community of individuals with depression |
|  |  | (-): healthy subjects were chosen from different populations with depression |
|  |  | (?): the information was unclear or not provided |
| Definition of Controls | 4 | (+): healthy controls did not report other psychiatric conditions and were evaluated with the same psychiatric questionnaires and interviews as depression. |
|  |  | (-): the information was not provided |
|  |  | (?): the mental disorders screening tool for healthy subjects was not described |
| **Comparability** | | |
| Age & Gender | 1 | (+): age and gender were matched between groups |
|  |  | (-): there was a significant difference in age and gender between groups |
|  |  | (?): the information was not provided |
| Other variables | 2 | (+): other demographic variable (e.g., education) were reported without significant differences among groups |
|  |  | (-): variables were reported with significant differences among groups |
|  |  | (?): the information was not provided |
| **Exposure** | | |
| Ascertain of Exposure | 1 | (+): same experimental procedure was performed in the group of depression patients and healthy controls |
|  |  | (-): experimental procedures were different among the groups |
|  |  | (?): the information was not provided |
| Drop-out | 2 | (+): studies reported the rate and causes of subjects’ drop-off |
|  |  | (-): the information was not provided |
|  |  | (?): the drop-out rate was reported but no explanation was given |
| Behavioral manipulation check | 3 | (+): behavioral task used in the experimental procedure led to a significant difference in patients as compared to healthy controls |
|  |  | (-): the results were not significantly different between the two groups |
|  |  | (?): the information was not provided |
| **Statistical inference** | | |
| Uncorrected p-value threshold | 1 | (+): studies reported an uncorrected p-value < 0.001 |
|  |  | (-): studies reported an uncorrected p-value＞0.001 |
|  |  | (?): the information was not provided |
| False positive corrections | 2 | (+): Studies described and performed false positive correction |
|  |  | (-): false positive correction was not performed or results not surviving after correction |
|  |  | (?): false positive correction was declared but not described |

**Table 3. Reasons for exclusion of full manuscripts screened and not included in systematic review**

| Manuscript | Reasons |
| --- | --- |
| 抑郁症情绪记忆中尾状核激活性别相关差异的fMRI研究 | 1 |
| Anticipation of aversive stimuli activates extended amygdala in unipolar depression | 1 |
| Elevated amygdala activity to sad facial expressions: a state marker of bipolar but not unipolar depression | 1 |
| Effects of Electroconvulsive Therapy on Brain Functional Activation and Connectivity in Depression | 1 |
| Common and distinct amygdala-function perturbations in depressed vs anxious adolescents  Neurofunctional Correlates of Response to Quetiapine in Adolescents with Bipolar Depression | 1 |
| Amygdala reactivity to masked negative faces is associated with automatic judgmental bias in major depression: a 3 T fMR1 study | 1 |
| Neural substrates for processing task-irrelevant emotional distracters in maltreated adolescents with depressive disorders: A pilot study | 1 |
| Internal reliability of blame-related functional MRI measures in major depressive disorder | 1 |
| Blunted amygdala activity is associated with depression severity in treatment-resistant depression | 1 |
| Emotional faces processing in major depressive disorder and prediction of antidepressant treatment response: A NeuroPharm study | 1 |
| Pretreatment Differences in BOLD Response to Emotional Faces Correlate with Antidepressant Response to Scopolamine | 1 |
| Association between depression severity and amygdala reactivity during sad face viewing in depressed preschoolers: an fMRI study | 1 |
| Differences in the neural correlates of affective responses in depressed and healthy women | 1 |
| Neural substrates of negativity bias in women with and without major depression | 1 |
| Childhood trauma history differentiates amygdala response to sad faces within MDD | 1 |
| Emotion-related brain activity to conflicting socio-emotional cues in unmedicated depression | 1 |
| Amygdala excitability to subliminally presented emotional faces distinguishes unipolar and bipolar depression: an fMRI and pattern classification study | 1 |
| An fMRI study of emotional face processing in adolescent major depression | 1 |
| Neural activation during facial emotion processing in unmedicated bipolar depression, euthymia, and mania | 1 |
| Effect of specific psychotherapy for chronic depression on neural Responses to emotional faces | 1 |
| Brain response to emotional faces in anxiety and depression: neural predictors of cognitive behavioral therapy outcome and predictor-based subgroups following therapy | 1 |
| Amygdala Activation and Connectivity to Emotional Processing Distinguishes Asymptomatic Patients With Bipolar Disorders and Unipolar Depression | 1 |
| The neural substrates of affective processing toward positive and negative affective pictures in patients with major depressive disorder | 1 |
| Neural correlates of affective processing in response to sad and angry facial stimuli in patients with major depressive disorder | 1 |
| The Course of Disease in Major Depressive Disorder Is Associated With Altered Activity of the Limbic System During Negative Emotion Processing | 1 |
| Dysfunction of fronto-limbic brain circuitry in depression | 1 |
| Neural substrates of trait ruminations in depression | 1 |
| Functional Brain Response to Emotional Musical Stimuli in Depression, Using INLA Approach for Approximate Bayesian Inference | 1 |
| CHANGES IN REGIONAL BRAIN ACTIVATION RELATED TO DEPRESSIVE STATE: A 2-YEAR LONGITUDINAL FUNCTIONAL MRI STUDY | 1 |
| Amygdala hyperactivation in untreated depressed individuals | 1 |
| Real-time fMRI of temporolimbic regions detects amygdala activation during single-trial self-induced sadness | 1 |
| Neural predictors and effects of cognitive behavioral therapy for depression: the role of emotional reactivity and regulation | 1 |
| Successful Pharmacologic Treatment of Major Depressive Disorder Attenuates Amygdala Activation to Negative Facial Expressions: A Functional Magnetic Resonance Imaging Study | 1 |
| Anterior cingulate cortex activity during attentional control corresponds with rumination in depression and social anxiety | 1 |
| Increased amygdala response to masked emotional faces in depressed subjects resolves with antidepressant treatment: An fMRI study | 1 |
| Can't shake that feeling: Assessment of sustained event-related fMRI amygdala activity in response to emotional information in depressed individuals | 1 |
| Increased association over time between regional frontal lobe BOLD change magnitude and cardiac vagal control with sertraline treatment for major depression | 1 |
| Altered functional connectivity between medial prefrontal cortex and the inferior brainstem in major depression during appraisal of subjective emotional responses: A preliminary study | 1 |
| Mood-congruent amygdala responses to subliminally presented facial expressions in major depression: associations with anhedonia | 1 |
| Early life stress and trauma and enhanced limbic activation to emotionally valenced faces in depressed and healthy children | 1 |
| Amygdala Response to Implicit and Explicit Sad Face Stimuli at Baseline Predicts Antidepressant Treatment Response to Scopolamine in Major Depressive Disorder | 1 |
| Amygdala response to explicit sad face stimuli at baseline predicts antidepressant treatment response to scopolamine in major depressive disorder | 1 |
| Altered Effective Connectivity within the Fronto-Limbic Circuitry in Response to Negative Emotional Task in Female Patients with Major Depressive Disorder | 1 |
| Functional magnetic resonance imaging correlates of emotion recognition and voluntary attentional regulation in depression: A generalized psycho physiological interaction study | 1 |
| BOLD activation of the ventromedial prefrontal cortex in patients with late life depression and comparison participants | 1 |
| Relationship Between Amygdala Responses to Masked Faces and Mood State and Treatment in Major Depressive Disorder | 1 |
| Changes in the neural correlates of implicit emotional face processing during antidepressant treatment in major depressive disorder | 1 |
| Imaging emotion circuits to predict treatment outcomes in major depressive disorder: the international study to predict optimized treatment in depression | 1 |
| Suicide Risk and Mood Regulation Deficits: Emotional Reactivity as an Exploratory Pathway | 2 |
| Neural Correlates of Impaired Removal of Negative Information From Working Memory are Associated With Rumination and Reappraisal in Individuals Diagnosed With Major Depressive Disorder | 2 |
| A neuroimaging study of functional connectivity during an emotion regulation task in major depressive disorder and borderline personality disorder | 2 |
| Effects of attention on emotional face processing in depression: a functional magnetic resonance imaging study | 2 |
| A Double Dissociation Between Anxiety and Depression Symptom Improvement and Fusiform Coupling During Positive and Negative Emotional Face Processing | 2 |
| Comorbid Anxiety, Anger, and Anhedonia as Moderators for Recruitment of Emotion Regulation Neurocircuitry in Major Depression | 2 |
| Influences of Anxiety and Depression on Emotional Processing and Its Implicit Regulation in Adults | 2 |
| Attenuation of Neural Activity During Emotion Processing in Unipolar and Bipolar Depression | 2 |
| Responses to Particular Negative Emotional Stimuli Discriminate Bipolar from Unipolar Depression | 2 |
| Differences in Emotional Attention Biases Between Suicide Attempters and Depressed Non-Attempters | 2 |
| Self-regulation of emotion: Modulation via coordinated dorso-rostral cingulate activity in MDD | 2 |
| Association between Deficits in Emotion Regulation (DERS) and Cingulate Function in MDD and Controls | 2 |
| Subjective Experience of Regulation of Emotion and Amgydgala Activity in MDD | 2 |
| Differential neural processing of social exclusion in adolescents with non-suicidal self-injury: An fMRI study | 2 |
| Clustering of emotional processing data in subjects with generalised anxiety disorder and major depression | 2 |
| Dynamic Functional Alterations of the Emotional Face Processing Network in Young People With Depression | 2 |
| Prefrontal regulation of the emotional brain: Findings in depressed and healthy subjects from neuroimaging and psychophysiology | 2 |
| Neural basis of the vulnerability to suicidal behavior: An fMRI study using a happy/angry faces paradigm | 2 |
| Functional Activation During Emotion Processing in Late-Life Depression: Early Markers of Treatment Response | 2 |
| Beyond "Concentration Difficulties": Probing Attention Impairments in Depression and Anxiety Across Multiple Units of Analysis | 2 |
| Anterior Cingulate Cortex Activity in Implicit and Explicit Emotion Regulation Predicts Cognitive Behavioral Therapy Response in Anxiety and Depression | 2 |
| Neurobiology of Processing Emotional Prosody in Unipolar Depression | 2 |
| The impact of autobiographical script driven imagery on emotion regulation and sustained brain activation in chronic depression | 2 |
| Neural Differences Between Euthymic Bipolar and Remitted Unipolar Depressed Individuals: An fMRI Study of Emotion Processing | 2 |
| Gender Differences in Brain Activation During Implicit Emotional Processing in Patients With Melancholic Depression | 2 |
| Associations between disease course and abnormal brain function during emotion processing in major depressive disorder | 2 |
| Associations between abnormal brain function during emotion processing and lifetime disease course in major depressive disorder | 2 |
| Gender Differences in the Neural Circuitry Underlying Cognitive Reappraisal of Emotional Stimuli in Adolescent Depression | 2 |
| Adolescent Depression is Associated with Reduced Structural Connectivity in Emotion Regulatory Circuitry | 2 |
| Fronto-striatal-thalamic Responses to Faces Associated with Suicide Attempts in Female Adolescents with Major Depressive Disorder | 2 |
| Neuropsychological impairment: the disturbed effect of self-processing in major depressive disorder | 2 |
| Effect of Ketamine Treatment on Amygdala Responsivity in Major Depressive Disorder | 2 |
| Neural Reactivity to Affective Social Stimuli in Generalized Anxiety, Social Anxiety, Major Depressive and Post-traumatic Stress Disorders: Disorder-specific and Transdiagnostic Associations | 2 |
| Functional magnetic resonance imaging (fMRI) of cognitive generation of affect in bipolar depression | 2 |
| Parietal Activation During Anticipation of Happy and Fear Faces is Related to Current Depression and Life-time Mania Across Diagnoses | 2 |
| Novel Transcriptome-Based Polygenic Risk Score for Molecular Vulnerability to Depression Predicts Brain Function During Face Processing | 2 |
| Amygdala activation is related to sympathovagal balance during emotional face matching in subjects with major depression | 2 |
| Cognitive Behavioral Therapy for Depression and the Neural Correlates of Emotion Regulation: Prediction of Treatment Outcome and Longitudinal Effects | 2 |
| Electroconvulsive Therapy Modulates Neural Response to Emotional Faces in Depressed Patients: A Randomized Controlled fMRI Study | 2 |
| Persisting neuropsychological cognitive-affective deficits in emotional processing during remission in recurrent major depressive disorder: associations with cognitive reactivity and future recurrence? | 2 |
| Early Life Adversity is Associated With Enhanced Fronto-Limbic Reactivity During Fear Processing in Adults With Depression and Anxiety | 2 |
| HPA-Axis Stress Reactivity Relates to the Degree of Prefrontal, Limbic, and Striatal Activation During Emotional Processing in Unipolar and Bipolar Depression | 2 |
| Differential patterns of neural response to sad versus happy facial expressions distinguish depressed from healthy individuals | 2 |
| Depression as a specific regime of brain functioning: results of neuroimaging studies | 2 |
| The neurobiology of self-processing in abused depressed adolescents | 2 |
| Functional MRI investigation using visual and emotional memory induction paradigms in chronic depression | 2 |
| Ketamine's effects on brain function during emo tional processing in major depressive disorder | 2 |
| Antidepressant Effects of Ketamine Versus Placebo are Differentially Associated With Brain Activity During Emotional Processing | 2 |
| A little emotion goes a long way: Increased amygdala responses to momentary emotional stimuli but blunted responses to repeated stimuli in unipolar depression | 2 |
| Relationships between amygdala volume and activity during emotional information processing tasks in depressed and never-depressed individuals - An fMRI investigation | 2 |
| Rostral cingulate activity is linked to problems in emotion regulation in the daily lives of adolescents with depression and anxiety | 2 |
| Emotion processing related brain activity assessed with 7 Tesla fMRI predicts early antidepressant response | 2 |
| Functional Neuroimaging of Emotion Regulation in Late-Life Depression | 2 |
| Baseline Neural Response During the Processing of Emotional Faces Predicts Antidepressant Response to Ketamine | 2 |
| Neural Processing of Emotional Stimuli in Visual Cortex Correlates with Antidepressant Response to Ketamine | 2 |
| Amygdala Response to Selective Emotion Processing Conditions at Baseline Predicts Antidepressant Treatment Response to Scopolamine in Major Depressive Disorder | 2 |
| Depressed Patients With History of Suicide Attempts Differ From Non-Attempters in Processing of Emotional Faces | 2 |
| Understanding neurocognitive working mechanisms of relapse prevention - the role of emotion regulation in remitted major depressive disorder | 2 |
| LONGITUDINAL CHANGES IN DEPRESSION PREDICT CHANGES IN MEDIAL PREFRONTAL CORTEX RESPONSE TO SAD FACES IN LATE ADOLESCENT GIRLS | 2 |
| Emotion regulation and functional magnetic resonance imaging in bipolar II depression vs. major depressive disorder | 2 |
| Depressed Patients Show fMRI Activity Alterations in Cognitive Control and Valuation Systems when Reappraising Negative Statements | 2 |
| Negative Emotion Biases; What's Common and What's not Across Measures and Dimensions of Depression and Anxiety | 2 |
| Imaging Emotion Circuits as Predictors of Treatment Outcomes in the ISPOT-D Study of Major Depression | 2 |
| Amygdala Activation to Emotion Stimuli as a Predictor of Treatment Outcomes in Major Depressive Disorder: The International Study to Predict Optimized Treatment in Depression (iSPOT-D) | 2 |
| A Neural Signature for Emotion Regulation in Depressed Adults: Associations With Real-World Management of Stress and Suicidal Ideation | 2 |
| Network of Regions Showing Stronger Connectivity During Emotion (versus identify) Working Memory Correlate with Antidepressant Response to Scopolamine | 2 |
| Disorder- and cognitive demand-specific neurofunctional alterations during social emotional working memory in generalized anxiety disorder and major depressive disorder | 3 |
| Pretreatment brain connectivity during positive emotion upregulation predicts decreased anhedonia following behavioral activation therapy for depression | 3 |
| [Changes in the emotional processing in depressive patients: a study with functional magnetoresonance tomography under the employment of pictures with affective contents] | 3 |
| Depression is associated with hyperconnectivity of an introspective socio-affective network during the recall of formative relationship episodes | 3 |
| Default mode network alterations during implicit emotional faces processing in first-episode, treatment-naive major depression patients | 3 |
| Neural correlates of depressive realism - An fMRI study on causal attribution in depression | 3 |
| Effective connectivity during face processing in major depression - distinguishing markers of pathology, risk, and resilience | 3 |
| Multimodal brain imaging connectivity analyses of emotional and motivational deficits in depression among women | 3 |
| Cortisol effects on brain functional connectivity during emotion processing in women with depression | 3 |
| Therapeutic mechanisms of psilocybin: Changes in amygdala and prefrontal functional connectivity during emotional processing after psilocybin for treatment-resistant depression | 3 |
| Decreased functional coupling of the amygdala and supragenual cingulate is related to increased depression in unmedicated individuals with current major depressive disorder | 3 |
| Altered amygdala circuits underlying valence processing among manic and depressed phases in bipolar adults | 3 |
| Neural correlates of treatment outcome in major depression | 3 |
| Integrating Multilevel Functional Characteristics Reveals Aberrant Neural Patterns during Audiovisual Emotional Processing in Depression | 3 |
| Shared facial emotion processing functional network findings in medication-naïve major depressive disorder and healthy individuals: detection by sICA | 3 |
| Functional connectivity between the amygdala and prefrontal cortex in medication-naive individuals with major depressive disorder | 3 |
| Alterations in the default mode-salience network circuit provide a potential mechanism supporting negativity bias in depression | 3 |
| Reversed frontotemporal connectivity during emotional face processing in remitted depression | 3 |
| Disrupted amygdala-prefrontal connectivity during emotion regulation links stress-reactive rumination and adolescent depressive symptoms | 3 |
| Aberrant default-mode network-hippocampus connectivity after sad memory-recall in remitted-depression | 3 |
| Abnormal left-sided orbitomedial prefrontal cortical-amygdala connectivity during happy and fear face processing: a potential neural mechanism of female MDD | 3 |
| Amygdala Functional Connectivity During Self-Face Processing in Depressed Adolescents With Recent Suicide Attempt | 3 |
| Opposing and emotion-specific associations between frontal activation with depression and anxiety symptoms during facial emotion processing in generalized anxiety and depression | 3 |
| Association of disease course and brain structural alterations in major depressive disorder | 4 |
| 重性抑郁症患者治疗后识别动态面部表情的fMRI研究 | 5 |
| Brain functional effects of electroconvulsive therapy during emotional processing in major depressive disorder | 5 |
| Anterior cingulate cortex activation during attentional control as a transdiagnostic marker of psychotherapy response: a randomized clinical trial | 5 |
| Different effects of mirtazapine and venlafaxine on brain activation: an open randomized controlled fMRI study | 5 |
| Neural Response to Implicit Emotions as Biomarkers of Clinical Response to SSRI Treatment in Depression | 5 |
| Emotion-based brain mechanisms and predictors for SSRI and CBT treatment of anxiety and depression: a randomized trial | 5 |
| The neural basis of cognitive and emotional processing in persistently depressed patients treated with desvenlafaxine | 5 |
| Brain functional changes in facial expression recognition in patients with major depressive disorder before and after antidepressant treatment: A functional magnetic resonance imaging study | 5 |
| Low-dose augmentation with buprenorphine increases emotional reactivity but not reward activity in treatment resistant mid- and late-life depression | 5 |
| fMRI response to negative words and SSRI treatment outcome in major depressive disorder: A preliminary study | 5 |
| Does a single session of electroconvulsive therapy alter the neural response to emotional faces in depression? A randomised sham-controlled functional magnetic resonance imaging study | 5 |
| Emotional recognition training modifies neural response to emotional faces but does not improve mood in healthy volunteers with high levels of depressive symptoms | 5 |
| Effects of electroconvulsive therapy on amygdala function in major depression - a longitudinal functional magnetic resonance imaging study | 5 |
| Effects of Ketamine on Brain Activity During Emotional Processing: Differential Findings in Depressed Versus Healthy Control Participants | 5 |
| Effect of bupropion extended release on negative emotion processing in major depressive disorder: a pilot functional magnetic resonance imaging study | 5 |
| Increased amygdala responses to emotional faces after psilocybin for treatment-resistant depression | 5 |
| Event-related functional magnetic resonance imaging measures of neural activity to positive social stimuli in pre- and post-treatment depression | 5 |
| Increased Reactivity of the Mesolimbic Reward System after Ketamine Injection in Patients with Treatment-resistant Major Depressive Disorder | 5 |
| Amygdala Reactivity to Emotional Faces in the Prediction of General and Medication-Specific Responses to Antidepressant Treatment in the Randomized iSPOT-D Trial | 5 |
| Excitatory brain stimulation over the left dorsolateral prefrontal cortex enhances voluntary distraction in depressed patients | 5 |
| Early post-treatment blood oxygenation level-dependent responses to emotion processing associated with clinical response to pharmacological treatment in major depressive disorder | 5 |
| 情绪调节的神经机制：从休息到习惯化 | 6 |
| Risk for depression is associated with neural biases in emotional categorisation | 6 |
| Neural mechanisms of subclinical depressive symptoms in women: a pilot functional brain imaging study | 6 |
| Blunted feelings: Alexithymia is associated with a diminished neural response to speech prosody | 6 |
| A dimensional approach to determine common and specific neurofunctional markers for depression and social anxiety during emotional face processing | 6 |
| Sex-related differences in neural activity during emotion regulation | 6 |
| Neural Correlates of Emotion Regulation and Adolescent Suicidal Ideation | 6 |
| Increased neural response to fear in patients recovered from depression: a 3T functional magnetic resonance imaging study | 6 |
| Is postnatal depression a distinct subtype of major depressive disorder? An exploratory study | 6 |
| Neurobiological Markers of Resilience to Depression Following Childhood Maltreatment: The Role of Neural Circuits Supporting the Cognitive Control of Emotion | 6 |
| An fMRI-study on semantic priming of panic-related information in depression without comorbid anxiety | 6 |
| The neural processing of negative emotion postpartum: a preliminary study of amygdala function in postpartum depression | 6 |
| Neural dysfunction in postpartum depression: an fMRI pilot study | 6 |
| Mood alters amygdala activation to sad distractors during an attentional task | 6 |
| Amygdala activation to sad pictures during high-field (4 tesla) functional magnetic resonance imaging | 6 |
| Dysfunction of emotional brain systems in individuals at high risk of mood disorder with depression and predictive features prior to illness | 6 |
| Randomized Clinical Trial of Real-Time fMRI Amygdala Neurofeedback for Major Depressive Disorder: Effects on Symptoms and Autobiographical Memory Recall | 6 |
| Amygdala and dorsomedial hyperactivity to emotional faces in youth with remitted Major Depression | 6 |
| 抑郁症患者外显性与内隐性情绪处理的脑功能磁共振研究 | 7 |
| Depression symptoms and cognitive control of emotion cues: a functional magnetic resonance imaging study | 7 |
| Bipolar I disorder and major depressive disorder show similar brain activation during depression | 7 |
| Explicit and implicit facial affect recognition in manic and depressed states of bipolar disorder: A functional magnetic resonance imaging study | 7 |
| Hyperactivation in Cognitive Control and Visual Attention Brain Regions During Emotional Interference in Adolescent Depression | 7 |
| Pattern classification of sad facial processing: Toward the development of neurobiological markers in depression | 7 |
| Altered negative BOLD responses in the default-mode network during emotion processing in depressed subjects | 7 |
| A pilot fMRI study of the effect of stressful factors on the onset of depression in female patients | 7 |
| BDNF Val66Met polymorphism in patterns of neural activation in individuals with MDD and healthy controls | 7 |
| Pattern recognition analyses of brain activation elicited by happy and neutral faces in unipolar and bipolar depression | 7 |
| Investigating neural circuits of emotion regulation to distinguish euthymic patients with bipolar disorder and major depressive disorder | 7 |
| Ketamine normalizes brain activity during emotionally valenced attentional processing in depression | 7 |
| Emotion processing in depression with and without comorbid anxiety disorder | 7 |
| Impulsive traits and unplanned suicide attempts predict exaggerated prefrontal response to angry faces in the elderly | 7 |
| Neural mechanisms of expectancy-based placebo effects in antidepressant clinical trials | 7 |
| 色氨酸羟化酶1和单胺氧化酶A基因对抑郁症患者额叶负性情绪加工功能的叠加效应 | 8 |
| 5-HTTLPR biases amygdala activity in response to masked facial expressions in major depression | 8 |
| Impaired attribution of emotion to facial expressions in anxiety and major depression | 8 |
| Regulation of Emotion in Major Depressive Disorder | 8 |
| Modulation of amygdala reactivity following rapidly acting interventions for major depression | 8 |
| Altered task modulation of global signal topography in the default-mode network of unmedicated major depressive disorder | 8 |
| Transdiagnostic neural correlates of affective face processing in anxiety and depression | 8 |
| Vulnerability for new episodes in recurrent major depressive disorder: protocol for the longitudinal DELTA-neuroimaging cohort study | 8 |
| Informing the study of suicidal thoughts and behaviors in distressed young adults: The use of a machine learning approach to identify neuroimaging, psychiatric, behavioral, and demographic correlates | 8 |
| Differential Patterns of Emotion Regulation Neural Circuitry Abnormalities Distinguish Bipolar and Unipolar Depression | 8 |
| Judgment of emotional information expressed by prosody and semantics in patients with unipolar depression | 8 |
| Neurocognitive working mechanisms of the prevention of relapse in remitted recurrent depression (NEWPRIDE): protocol of a randomized controlled neuroimaging trial of preventive cognitive therapy | 8 |
| Brain Response of Major Depressive Disorder Patients to Emotionally Positive and Negative Music | 8 |
| 首发与复发抑郁症患者识别悲伤面部表情的脑功能差异研究 | 9 |
| 肾阳亏虚型抑郁症患者面部动态情绪识别的脑功能研究 | 9 |
| 女性重性抑郁症患者识别动态面部表情的神经基础的fMRI研究 | 9 |
| 抑郁症患者对正性面部表情功能识别 | 9 |
| Shifted inferior frontal laterality in women with major depressive disorder is related to emotion-processing deficits | 9 |
| Neuronal correlates of emotional processing in patients with major depression | 9 |
| Emotion-Dependent Functional Connectivity of the Default Mode Network in Adolescent Depression | 9 |
| Effects of an antidepressant on neural correlates of emotional processing in patients with major depression | 9 |
| Neural Correlates of Dynamic Facial Expression Recognition in Female Patients with Major Depressive Disorder: a Functional Magnetic Resonance Study | 9 |
| Amygdala hyperactivation and prefrontal hypoactivation in subjects with cognitive vulnerability to depression | 9 |
| The neural correlates of emotional face-processing in adolescent depression: a dimensional approach focusing on anhedonia and illness severity | 10 |
| Longitudinal brain changes in MDD during emotional encoding: effects of presence and persistence of symptomatology | 11 |
| Brain Activation During Emotional Memory Processing Associated with Subsequent Course of Depression | 11 |
| Differential relations of suicidality in depression to brain activation during emotional and executive processing | 11 |
| Reduced accuracy accompanied by reduced neural activity during the performance of an emotional conflict task by unmedicated patients with major depression: A CAN-BIND fMRI study | 11 |
| Escitalopram ameliorates differences in neural activity between healthy comparison and major depressive disorder groups on an fMRI Emotional conflict task: A CAN-BIND-1 study | 11 |
| Increased amygdala responses to sad but not fearful faces in major depression: relation to mood state and pharmacological treatment | 11 |
| Music in depression: Neural correlates of emotional experience in remitted depression | 11 |
| The functional neuroanatomy of major depression: an fMRI study using an emotional activation paradigm | 11 |
| Brain activation to emotional words in depressed vs healthy subjects | 11 |
| Neuroimaging correlates of emotional response-inhibition discriminate between young depressed adults with and without sub-threshold bipolar symptoms | 11 |
| Brain circuitries involved in emotional interference task in major depression disorder | 11 |
| The attention biases to emotional stimulus in depressive patients and the evidence from functional magnetic resonance imaging | 11 |
| Brain imaging correlates of depressive symptom severity and predictors of symptom improvement after antidepressant treatment | 11 |
| Transcranial Direct Current Stimulation Over the Prefrontal Cortex in Depression Modulates Cortical Excitability in Emotion Regulation Regions as Measured by Concurrent Functional Magnetic Resonance Imaging: An Exploratory Study | 11 |
| Aberrant brain responses to emotionally valent words is normalised after cognitive behavioural therapy in female depressed adolescents | 11 |
| Neural Aspects of Inhibition Following Emotional Primes in Depressed Adolescents | 11 |
| Orbito-frontal cortex mechanism of inhibition of return in current and remitted depression | 11 |
| Dopamine D-3 receptor gene variation: impact on electroconvulsive therapy response and ventral striatum responsiveness in depression | 11 |
| The neural substrates of affective processing in depressed patients treated with venlafaxine | 11 |
| Neural correlates of top-down regulation and generation of negative affect in major depressive disorder | 11 |
| An fMRI study of cognitive reappraisal in major depressive disorder and borderline personality disorder | 11 |
| Antidepressant short-term and long-term brain effects during self-referential processing in major depression | 11 |
| Neural correlates of perception of emotional facial expressions in out-patients with mild-to-moderate depression and anxiety. A multicenter fMRI study | 11 |
| Affective context interferes with cognitive control in unipolar depression: An fMRI investigation | 11 |
| Altered Neural Function During Episodic Memory Encoding and Retrieval in Major Depression | 11 |
| Neural activity to intense positive versus negative stimuli can help differentiate bipolar disorder from unipolar major depressive disorder in depressed adolescents: a pilot fMRI study | 11 |
| Evidence of successful modulation of brain activation and subjective experience during reappraisal of negative emotion in unmedicated depression | 11 |
| Negative Autobiographical Memory in Depression Reflects Elevated Amygdala-Hippocampal Reactivity and Hippocampally Associated Emotion Regulation | 11 |
| Ventromedial prefrontal cortex and amygdala dysfunction during an anger induction positron emission tomography study in patients with major depression disorder with anger attacks | 11 |
| Reduced medial prefrontal responses to social interaction images in remitted depression | 11 |
| Acute and sustained effects of cognitive emotion regulation in major depression | 11 |
| Common abnormalities and disorder-specific compensation during implicit regulation of emotional processing in generalized anxiety and major depressive disorders | 11 |
| Altered emotional interference processing in affective and cognitive-control brain circuitry in major depression | 11 |
| Mood-linked responses in medial prefrontal cortex predict relapse in patients with recurrent unipolar depression | 11 |
| Self-other referential neural processing in social anxiety disorder and major depressive disorder | 11 |
| Transdiagnostic neural correlates of volitional emotion regulation in anxiety and depression | 11 |
| Recalling happy memories in remitted depression: A neuroimaging investigation of the repair of sad mood | 11 |
| Activation of the medial prefrontal and posterior cingulate cortex during encoding of negative material predicts symptom worsening in major depression | 11 |
| Within- and Between-Session Changes in Neural Activity During Emotion Processing in Unipolar and Bipolar Depression | 11 |
| Amygdala and whole-brain activity to emotional faces distinguishes major depressive disorder and bipolar disorder | 11 |
| Multimodal functional and structural neuroimaging investigation of major depressive disorder following treatment with duloxetine | 11 |
| Neural responses to happy facial expressions in major depression following antidepressant treatment | 11 |
| Attenuation of the neural response to sad faces in major depression by antidepressant treatment - A prospective, event-related functional magnetic resonance imaging study | 11 |
| Neural responses to sad facial expressions in major depression following cognitive behavioral therapy | 11 |
| Early changes in emotional processing as a marker of clinical response to SSRI treatment in depression | 11 |
| Subgenual anterior cingulate activation to valenced emotional stimuli in major depression | 11 |
| The neural correlates of regulating positive and negative emotions in medication-free major depression | 11 |
| Reduced negative BOLD responses in the default-mode network and increased self-focus in depression | 11 |
| Increased Self-Focus in Major Depressive Disorder Is Related to Neural Abnormalities in Subcortical-Cortical Midline Structures | 11 |
| Discriminating unipolar and bipolar depression by means of fMRI and pattern classification: a pilot study | 11 |
| Neural correlates of causal attribution in negative events of depressed patients: Evidence from an fMRI study | 11 |
| The neural correlates of emotional face-processing in adolescent depression: a dimensional approach focusing on anhedonia and illness severity | 11 |
| Altered insular activation and increased insular functional connectivity during sad and happy face processing in adolescent major depressive disorder | 11 |
| Functional Correlates of childhood maltreatment and symptom severity during affective theory of mind tasks in chronic depression | 11 |
| Emotion introspection and regulation in depression | 11 |
| Functional connectivity of negative emotional processing in adolescent depression | 11 |
| Functional MRI of emotional memory in adolescent depression | 11 |
| The effects of catecholamine depletion on the neural response to fearful faces in remitted depression | 11 |
| fMRI BOLD responses to negative stimuli in the prefrontal cortex are dependent on levels of recent negative life stress in major depressive disorder | 11 |
| Cognitive reserve-mediated neural modulation of emotional control and regulation in people with late-life depression | 11 |
| Orbitofrontal cortex response to angry faces in men with histories of suicide attempts | 11 |
| Auditory processing in remitted major depression: a long-term follow-up investigation using 3T-fMRI | 11 |
| Distracted and down: neural mechanisms of affective interference in subclinical depression | 11 |
| Functional magnetic resonance imaging studies of emotional processing in normal and depressed patients: Effects of venlafaxine | 11 |
| Neural correlates of emotion regulation deficits in remitted depression: the influence of regulation strategy, habitual regulation use, and emotional valence | 11 |
| Neural markers of symptomatic improvement during antidepressant therapy in severe depression: subgenual cingulate and visual cortical responses to sad, but not happy, facial stimuli are correlated with changes in symptom score | 11 |
| A double dissociation of ventromedial prefrontal cortical responses to sad and happy stimuli in depressed and healthy individuals | 11 |
| Subgenual cingulate and visual cortex responses to sad faces predict clinical outcome during antidepressant treatment for depression | 11 |
| Transdiagnostic alterations in neural emotion regulation circuits - neural substrates of cognitive reappraisal in patients with depression and post-traumatic stress disorder | 11 |
| Altered Functional Magnetic Resonance Imaging Markers of Affective Processing During Treatment of Late-Life Depression | 11 |
| Depressed mood and lateralized prefrontal activity during a Stroop task in adolescent children | 11 |
| Brain response to emotional faces in anxiety and depression: neural predictors of cognitive behavioral therapy outcome and predictor-based subgroups following therapy | 11 |
| Short-term escitalopram treatment normalizes aberrant self-referential processing in major depressive disorder | 11 |
| Using standardized fMRI protocols to identify patterns of prefrontal circuit dysregulation that are common and specific to cognitive and emotional tasks in major depressive disorder: first wave results from the iSPOT-D study | 11 |
| Neural abnormalities during cognitive generation of affect in treatment-resistant depression | 11 |
| Neurocognitive Measures of Self-blame and Risk Prediction Models of Recurrence in Major Depressive Disorder | 11 |
| Subcortical and ventral prefrontal cortical neural responses to facial expressions distinguish patients with bipolar disorder and major depression | 11 |
| A study of emotional processing in unipolar depression using fMRI | 11 |
| Self-referential processing in unipolar depression: Distinct roles of subregions of the medial prefrontal cortex | 11 |
| Reduced right ventrolateral prefrontal cortex activity while inhibiting positive affect is associated with improvement in hedonic capacity after 8 weeks of antidepressant treatment in major depressive disorder | 11 |
| Disorder- and emotional context-specific neurofunctional alterations during inhibitory control in generalized anxiety and major depressive disorder | 11 |
| Self-compassion and dorsolateral prefrontal cortex activity during sad self-face recognition in depressed adolescents | 11 |
| The regulation of positive and negative emotions through instructed causal attributions in lifetime depression - A functional magnetic resonance imaging study | 11 |
| Attention control and its emotion-specific association with cognitive emotion regulation in depression | 11 |
| Cognitive generation of affect in bipolar depression: an fMRI study | 11 |
| fMRI Response of Parietal Brain Areas to Sad Facial Stimuli in Mild Depression | 11 |
| Neural Response After a Single ECT Session During Retrieval of Emotional Self-Referent Words in Depression: A Randomized, Sham-Controlled fMRI Study | 11 |
| Neural response to pleasant stimuli in anhedonia: an fMRI study | 11 |
| Functional connectivity of the amygdala and subgenual cingulate during cognitive reappraisal of emotions in children with MDD history is associated with rumination | 11 |
| Processing of music in the first episode of major depressive disorder without treatment | 11 |
| The experience of social exclusion in women with a history of suicidal acts: a neuroimaging study | 11 |
| Anomalous functional brain activation following negative mood induction in children with pre-school onset major depression | 11 |
| Differential patterns of activity and functional connectivity in emotion processing neural circuitry to angry and happy faces in adolescents with and without suicide attempt | 11 |
| Neural and behavioural correlates of autobiographical memory retrieval in patients with major depressive disorder and a history of trauma exposure | 11 |
| Amygdala response and functional connectivity during emotion regulation: a study of 14 depressed adolescents | 11 |
| Cognitive reappraisal of peer rejection in depressed versus non-depressed adolescents: functional connectivity differences | 11 |
| The neurobiology of self face recognition among depressed adolescents | 11 |
| Neurofeedback and neuroplasticity of visual self-processing in depressed and healthy adolescents: A preliminary study | 11 |
| The neurobiology of self-knowledge in depressed and self-injurious youth | 11 |
| The neurobiology of self-face recognition in depressed adolescents with low or high suicidality | 11 |
| Imaging the up's and down's of emotion regulation in lifetime depression | 11 |
| Neural responses to dynamic multimodal stimuli and pathology-specific impairments of social cognition in schizophrenia and depression | 11 |
| Neural correlates of self-referential processing and implicit self-associations in chronic depression | 11 |
| Neural response to emotional stimuli associated with successful antidepressant treatment and behavioral activation | 11 |
| Functional neuroanatomy of emotion processing in major depressive disorder is altered after successful antidepressant therapy | 11 |
| Medial prefrontal cortex activity during memory encoding of pictures and its relation to symptomatic improvement after citalopram treatment in patients with major depression | 11 |
| Longitudinal effects of cognitive behavioral therapy for depression on the neural correlates of emotion regulation | 11 |
| Detached empathic experience of others' pain in remitted states of depression - An fMRI study | 11 |
| Brain activation predicts treatment improvement in patients with major depressive disorder | 11 |
| Other race effect on amygdala response during affective facial processing in major depression | 11 |
| The influence of positive and negative emotional associations on semantic processing in depression: an fMRI study | 11 |
| Use of FMRI to predict recovery from unipolar depression with cognitive behavior therapy | 11 |
| Increased amygdala and decreased dorsolateral prefrontal BOLD responses in unipolar depression: Related and independent features | 11 |
| Disentangling introspective and exteroceptive attentional control from emotional appraisal in depression using fMRI: A preliminary study | 11 |
| Neural indicators of emotion regulation via acceptance vs reappraisal in remitted major depressive disorder | 11 |
| Neural mechanisms of cognitive reappraisal in remitted major depressive disorder | 11 |
| Hard to look on the bright side: neural correlates of impaired emotion regulation in depressed youth | 11 |
| Feeling addressed! The neural processing of social communicative cues in patients with major depression | 11 |
| A differential pattern of neural response toward sad versus happy facial expressions in major depressive disorder | 11 |
| Depression is associated with increased sensitivity to signals of disgust: a functional magnetic resonance imaging study | 11 |
| Automatic mood-congruent amygdala responses to masked facial expressions in major depression | 11 |
| Structural-functional correlations between hippocampal volume and cortico-limbic emotional responses in depressed children | 11 |
| Brain Activity in Adollescent Major Depressive Disorder Before and After Fluoxetine Treatment | 11 |
| Interaction between a history of depression and rumination on neural response to emotional faces | 11 |
| Amygdala response to fearful faces in anxious and depressed children | 11 |
| Neuronal response to high negative affective stimuli in major depressive disorder: An fMRI study | 11 |
| Neural substrates of emotional conflict with anxiety in major depressive disorder: Findings from the Establishing Moderators and biosignatures of Antidepressant Response in Clinical Care (EMBARC) randomized controlled trial | 11 |
| Brain Responses to Emotional Stimuli after Eicosapentaenoic Acid and Docosahexaenoic Acid Treatments in Major Depressive Disorder: Toward Personalized Medicine with Anti-Inflammatory Nutraceuticals | 11 |
| Amygdala activation during emotional face processing in adolescents with affective disorders: the role of underlying depression and anxiety symptoms | 11 |
| Neural basis of positive and negative emotion regulation in remitted depression | 11 |
| Functional Magnetic Resonance Imaging Correlates of Emotional Word Encoding and Recognition in Depression and Anxiety Disorders | 11 |
| Neural state and trait bases of mood-incongruent memory formation and retrieval in first-episode major depression | 11 |
| The extended functional neuroanatomy of emotional processing biases for masked faces in major depressive disorder | 11 |
| Depressive state- and disease-related alterations in neural responses to affective and executive challenges in geriatric depression | 11 |
| Prefrontal mechanisms for executive control over emotional distraction are altered in major depression | 11 |
| Dissociable self effects for emotion regulation: a study of Chinese major depressive outpatients | 11 |
| The Role of Motivation in Cognitive Reappraisal for Depressed Patients | 11 |
| Brain activity in patients with deficiency versus excess patterns of major depression: A task fMRI study | 11 |
| Autobiographical memory in depression: An fMRI study | 11 |
| Incidental retrieval of emotional contexts in post-traumatic stress disorder and depression: An fMRI study | 11 |
| Cognitive behavioral therapy for depression changes medial prefrontal and ventral anterior cingulate cortex activity associated with self-referential processing | 11 |
| Behavioral and Neurophysiological Correlates of Autobiographical Memory Deficits in Patients With Depression and Individuals at High Risk for Depression | 11 |
| Functional neuroimaging correlates of autobiographical memory deficits in subjects at risk for depression | 11 |
| Differential neural correlates of autobiographical memory recall in bipolar and unipolar depression | 11 |
| Functional neuroimaging of sex differences in autobiographical memory recall in depression | 11 |
| Response to SSRI intervention and amygdala activity during self-referential processing in major depressive disorder | 11 |
| Real-Time Functional Magnetic Resonance Imaging Amygdala Neurofeedback Changes Positive Information Processing in Major Depressive Disorder | 11 |
| Amygdala Activity During Autobiographical Memory Recall in Depressed and Vulnerable Individuals: Association With Symptom Severity and Autobiographical Overgenerality | 11 |
| Real-Time fMRI Neurofeedback Training of Amygdala Activity in Patients with Major Depressive Disorder | 11 |
| Abnormal emotional and neural responses to romantic rejection and acceptance in depressed women | 11 |
| Different activation pattern to unattended emotional words in depressed vs.healthy subjects | 11 |
| In search of the depressive self: extended medial prefrontal network during self-referential processing in major depression | 11 |
| Recognition of Facial Emotion Expressions in Patients with Depressive Disorders: A Functional MRI Study | 11 |
| An exploratory examination of reappraisal success in depressed adolescents: Preliminary evidence of functional differences in cognitive control brain regions | 11 |
| Blunted superior temporal gyrus activity to negative emotional expression after mindfulness-based cognitive therapy for late-life depression | 11 |
| Reasons for exclusion classified as: 1. ROI-based studies; 2. Meeting abstract; 3. Different analyse methods; 4. Different detection methods; 5. Intervention studies; 6. Not depression patients; 7. Not relevant control group; 8. Not brain-imaging study; 9. Reduplicate articles; 10. No data available; 11. Different task type.  **Table 4. The details of fMRI acquisition and analysis** | |

| **Study** | MRI acquisition | | | | T1 | | | | T2 | | | | | Analysis | |  |
| --- | --- | --- | --- | --- | --- | --- | --- | --- | --- | --- | --- | --- | --- | --- | --- | --- |
|  | Teslas | MRI-system | MRI-model | Head-coil | Sequence | TR (ms) | TE (ms) | Voxel size (mm) | | Sequence | TR (ms) | TE (ms) | Voxel size (mm) | | Software | |
| van Wingen, 2011 | 1.5T | Siemens | Sonata | standard | MPRAGE | － | － | 1×1×1 | | EPI | 2290 | 30 | － | | SPM5 | |
| Townsend, 2010 | 3T | Siemens | Allegra | single-channel | － | － | － | － | | EPI | 2500 | 35 | 3.75×3.75×3 | | FSL | |
| Schlund, 2012 | 3T | Phillips | Sonata | 8-channel | MPRAGE | － | － | 1×1×1 | | EPI | 3000 | 35 | 3×3×3 | | SPM | |
| Ritchey, 2011 | 1.5T | GE | Signa | － | － | － | － | － | | spiral gradient-echo | 2000 | 40 | 3.75×3.75×5 | | SPM2 | |
| Nagy, 2021 | 3T | Siemens | Trio | 12- channel | MPRAGE | 2530 | 3.37 | － | | EPI | 2500 | 30 | － | | FSL | |
| Skokauskas,2015 | 3T | Philips | Achieva | － | SPGR | 8.5 | 3.9 | 1×1×1 | | SE-EPI | 2000 | 35 | 3×3×4.8 | | SPM8 | |
| Murrough, 2015 | 3T | Philips | Achieva | 8-channel | threedimensional turbo field echo | 7.5 | 3.5 | 1×1×1 | | EPI | 2000 | 26.6 | 2.2×2.2×2.5 | | SPM8 | |
| Zhong, 2012 | 1.5T | Siemens | － | standard | － | － | － | － | | EPI | 3000 | 40 | 3.8×3.8×5 | | SPM8 | |
| Mel'nikov, 2018 | 3T | Philips | Ingenia | － | TFE | － | － | 1×1×1 | | Ssh EPI | 2500 | 35 | 2×2×5 | | SPM8 | |
| Li, 2013 | 3T | Siemens | Trio | － | － | － | － | － | | － | 2000 | 30 | 3.5×3.5×3 | | SPM8 | |
| Groves, 2018 | 3T | GE | HDxt | 8-channel | three-dimensional spoiled gradient recalled echo | 6.7 | 2.8 | 0.98× 0.98 ×1 | | EPI | 2500 | 35 | 3.7×3.7×4 | | SPM12 | |
| Bürger, 2017 | 3T | Philips | － | － | － | － | － | － | | EPI | 2100 | 30 | 3.6 × 3.6 × 3.6 | | － | |
| Scheuerecker, 2010 | 3T | GE | Signa | － | 3-dimensional fast spoiled gradient echo | 6.9 | 3.2 | － | | EPI | 2100 | 35 | － | | SPM5 | |
| Derntl, 2011 | 3T | Siemens | Trio | standard | MPRAGE | 1900 | 2.52 | － | | EPI | 2200 | 30 | 3.1×3.1×3.1 | | SPM5 | |
| Bian, 2013 | 3T | Siemens | Trio | 12-channel | － | 350 | 2.5 | － | | EPI | 3000 | 30 | 3.1×3.1×3 | | SPM8 | |
| Cai, 2016 | 3T | Philips | － | standard | － | － | － | － | | EPI | 3000 | 30 | － | | SPM8 | |
| Yao, 2008 | 1.5T | GE | Signa | standard | Tlflait | 2000 | 7.3 | － | | GRE- EPI | 3000 | 40 | － | | SPM2 | |
| Cao, 2008 | 1.5T | GE | Signa | － | － | 2000 | 7.3 | － | | GRE- EPI | 3000 | 40 | － | | SPM2 | |
| Li, 2007 | 1.5T | GE | Signa | 8-channel | － | 2100 | 8 | － | | － | 4500 | 100 | － | | SPM2 | |
| Song, 2019 | 3T | Siemens | － | － | － | 1900 | 3.82 | － | | GRE- EPI | 2000 | 30 | － | | SPM8 | |
| Tu, 2012 | 3T | Siemens | － | standard | SE | 500 | 14 |  | | GRE- EPI | 2000 | 40 | － | | AFNI | |
| Xu, 2017 | 3T | Siemens | － | － | － | 3000 | 45 | 1×1×1 | | EPI | 2000 | 30 | 3×3×3 | | SPM8 | |
| Briceño, 2015 | － | － | － | － | － | － | － | － | | － | － | － | － | | － | |
| Yang, 2010 | 3T | GE | － | 8-channel | fast spoiled gradient echo | 8 | 3.1 | 0.98×0.98×1 | | EPI | 2000 | 32 | － | | AFNI | |
| Li, 2022 | 3T | GE | MR750 | 8-channel | － | － | － | － | | EPI | 2000 | 30 | － | | SPM12 | |
| Koch,2018 | 3T | Siemens | － | 20-channel | － | 2.3 | 4.16 | 1×1×1 | | － | 1500 | 34 | 2×2×2 | | SPM12 | |
| Lisiecka, 2013 | 3T | Philips | Achieva | － | spoiled gradient recalled acquisition | 8.5 | 3.9 | 1×1×1 | | EPI | 2000 | 35 | 3×3×4.8 | | SPM8 | |
| Ho, 2016 | 3T | GE | MR750 | 8-channel | fast spoiled gradient recalled | 8.1 | 3.17 | 0.98×0.98×1 | | EPI | 1000 | 30 | 3×3×3 | | AFNI | |
| MRI, magnetic resonance imaging; GE, gradient echo pulse; FSPGR, fast spoiled gradient sequence; MPRAGE, magnetization-prepared rapid acquisition with gradient echo sequence; SPGR, spoiled gradient recalled sequence; SPM, statistical parametric mapping; TR, Repetition time for the whole pulse sequence in MRI; TE, Echo time i.e. Time between middle of exciting Radio Frequency pulse and middle of spin echo production | | | | | | | | | | | | | | | | |

**Table 5. Risk of bias**

| **study** | **Selection** | | | | **Comparability** | | **Exposure** | | | **Statistical analysis** | |
| --- | --- | --- | --- | --- | --- | --- | --- | --- | --- | --- | --- |
|  |  | ② | **③** | **④** | **⑤** | **⑥** | **⑦** | **⑧** | **⑨** | ⑩ | ⑪ |
| van Wingen, 2011 | + | + | + | + | + | + | + | + | + | - | + |
| Townsend, 2010 | + | + | + | ? | + | ? | + | + | + | - | + |
| Schlund, 2012 | + | + | ? | + | ? | ? | + | - | + | + | - |
| Ritchey, 2011 | + | + | ? | ? | + | ? | + | + | + | ? | - |
| Nagy, 2021 | + | + | + | + | + | + | + | - | + | ? | + |
| Skokauskas,2015 | + | + | - | + | + | + | + | - | + | ? | + |
| Murrough, 2015 | + | + | - | - | + | ? | + | ? | + | - | + |
| Zhong, 2012 | + | + | ? | + | + | ? | + | + | + | - | - |
| Mel'nikov, 2018 | + | ? | ? | ? | + | + | + | - | + | + | + |
| Li, 2013 | + | + | + | ? | + | + | + | + | + | + | - |
| Groves, 2018 | + | + | + | + | ? | ? | + | + | + | + | - |
| Bürger, 2017 | + | + | + | + | + | + | + | + | + | ? | ? |
| Scheuerecker, 2010 | + | + | ? | ? | + | + | + | + | + | + | - |
| Derntl, 2011 | + | + | - | - | + | + | + | - | + | ? | + |
| Bian, 2013 | + | - | ? | + | + | + | + | ? | + | - | - |
| Cai, 2016 | + | + | + | + | + | + | + | + | + | - | + |
| Yao, 2008 | + | - | + | ? | + | + | + | - | + | + | - |
| Cao, 2008 | + | - | ? | + | + | + | + | - | + | + | - |
| Li, 2007 | + | + | ? | + | + | + | + | + | + | ? | - |
| Song, 2019 | + | + | ? | + | + | + | + | - | + | ? | + |
| Tu, 2012 | + | + | - | + | + | + | + | - | + | + | + |
| Xu, 2017 | + | + | + | + | + | + | + | - | + | ? | ? |
| Briceño, 2015 | + | + | + | ? | + | + | + | + | + | ? | + |
| Yang, 2010 | + | + | + | + | + | + | + | - | + | - | + |
| Li, 2022 | + | + | - | + | + | + | + | - | + | ? | + |
| Koch,2018 | + | + | + | + | + | + | + | - | + | ? | + |
| Lisiecka, 2013 | + | + | - | + | + | + | + | - | + | ? | + |
| Ho, 2016 | + | + | + | + | + | + | + | - | + | ? | + |
| **①adequate case definition; ②representativeness; ③controls; ④definition of controls; ⑤age and gender; ⑥other variables; ⑦same exposure; ⑧drop-out rate; ⑨behavioral manipulation check; ⑩p value > 0.001;** ⑪**false positive correction** | | | | | | | | | | | |

**Table 6. Results of sensitive analysis**

| **Discarded Article** | **Decreased** | | | | **Increased** | | | |
| --- | --- | --- | --- | --- | --- | --- | --- | --- |
|  | **Superior Temporal Gyrus** | **Inferior Parietal Lobule** | **Insula** | **Superior Frontal Gyrus** | **Middle Temporal Gyrus** | **Superior Temporal Gyrus** | **Parahippocampal Gyrus** | **Cuneus** |
| van Wingen, 2011 | Y | Y | Y | Y | Y | Y | Y | Y |
| Townsend, 2010 | Y | Y | Y | Y | Y | Y | Y | Y |
| Schlund, 2012 | Y | Y | Y | Y | Y | Y | Y | N |
| Ritchey, 2011 | Y | Y | Y | Y | Y | Y | Y | Y |
| Nagy, 2021 | Y | Y | Y | Y | Y | Y | Y | Y |
| Skokauskas,2015 | Y | N | Y | Y | Y | Y | Y | Y |
| Murrough, 2015 | Y | Y | Y | Y | Y | Y | Y | Y |
| Zhong, 2012 | Y | Y | Y | Y | Y | Y | Y | Y |
| Mel'nikov, 2018 | Y | Y | N | Y | Y | Y | Y | Y |
| Li, 2013 | Y | Y | Y | Y | Y | Y | Y | Y |
| Groves, 2018 | Y | Y | Y | Y | Y | Y | Y | Y |
| Bürger, 2017 | Y | Y | Y | Y | Y | Y | Y | Y |
| Scheuerecker, 2010 | Y | Y | Y | Y | Y | Y | Y | Y |
| Derntl, 2011 | Y | Y | Y | Y | Y | Y | Y | Y |
| Bian, 2013 | Y | Y | Y | Y | Y | Y | Y | N |
| Cai, 2016 | Y | Y | Y | Y | Y | Y | Y | Y |
| Yao, 2008 | Y | Y | Y | Y | N | N | Y | Y |
| Cao, 2008 | Y | Y | Y | Y | Y | Y | N | Y |
| Li, 2007 | Y | Y | Y | Y | N | N | Y | Y |
| Song, 2019 | Y | Y | Y | Y | Y | Y | Y | Y |
| Tu, 2012 | Y | Y | N | N | Y | Y | Y | Y |
| Xu, 2017 | Y | Y | Y | Y | Y | Y | Y | N |
| Briceño, 2015 | Y | Y | Y | Y | Y | Y | Y | Y |
| Yang, 2010 | Y | Y | Y | Y | Y | Y | Y | Y |
| Li, 2022 | Y | Y | Y | N | Y | Y | Y | Y |
| Koch,2018 | Y | Y | Y | Y | Y | Y | Y | Y |
| Lisiecka, 2013 | Y | N | N | Y | Y | Y | Y | Y |
| Ho, 2016 | Y | Y | Y | Y | Y | Y | Y | Y |
| Y: YES; N: NO | | | | | | | | |
